# A pre-emptive risk model for acute rejection in liver transplantation: an immunopharmacologic biomarker panel combining CD4+ T-cell profiling and tacrolimus exposure

**DOI:** 10.3389/fimmu.2026.1760409

**Published:** 2026-03-03

**Authors:** Qin-Xin Li, Jun-Xi Zhang, Han Li, Xian-Liang Li, Qiang He, Dong-Dong Han, Ji-Qiao Zhu

**Affiliations:** 1 Department of Hepatobiliary and Pancreaticosplenic Surgery, Medical Research Center, Beijing Organ Transplant Center, Beijing Chaoyang Hospital, Capital Medical University, Beijing, China; 2 Department of Head and Neck Surgery, National Cancer Center/National Clinical Research Center for Cancer/Cancer Hospital, Chinese Academy of Medical Sciences and Peking Union Medical College, Beijing, China; 3 Department of Hepatobiliary Surgery, China-Japan Friendship Hospital, Beijing, China

**Keywords:** acute rejection, liver transplantation, personalized medicine, predictive model, tacrolimus, therapeutic drug monitoring, CD4^+^ T-cell

## Abstract

**Introduction:**

Acute cellular rejection (ACR) is a T cell-driven event in liver transplantation. Current monitoring relies on detecting graft injury, lacking tools for pre-emptive risk assessment based on the patient’s real-time immune status.

**Methods:**

We developed an immunopharmacologic risk model in a retrospective cohort of 98 liver transplant recipients (18 with biopsy-proven ACR). The model integrated peripheral CD4+ T-cell percentage (flow cytometry) and tacrolimus trough level. Firth-penalized logistic regression was used for model development, with internal validation via bootstrapping.

**Results:**

The parsimonious model, comprising only CD4+ T-cell percentage and tacrolimus level, demonstrated good discrimination (AUC 0.774, 95% CI 0.674-0.874) and calibration. Critically, lead-time analysis revealed the model provided a median warning window of 8 days (IQR: 3.5 days) prior to biochemical injury onset. It offered significant incremental value over monitoring tacrolimus alone (AUC 0.774 vs. 0.694, ΔAUC=0.080, p=0.007) or CD4+ T cells alone (AUC 0.774 vs. 0.733, ΔAUC=0.041, p=0.014).

**Conclusion:**

We identify and validate a novel, clinically actionable immunopharmacologic biomarker panel for ACR. This model enables pre-emptive risk stratification by capturing the high-risk confluence of immune activation and subtherapeutic immunosuppression, paving the way for personalized immunotherapy in transplant recipients.

## Introduction

Liver transplantation has evolved into a life-saving therapy for end-stage liver disease, yet acute cellular rejection (ACR) remains an important clinical event that complicates post-transplant management and contributes to healthcare costs, occurring in up to 40% of recipients (1, 2). The pathogenesis of ACR represents a classic drug-disease interaction, orchestrated by a CD4+ T cell-centric inflammatory cascade (3, 4). Central to this process is the pharmacologic action of calcineurin inhibitors (CNIs) like tacrolimus. Subtherapeutic CNI levels fail to suppress nuclear factor of activated T cells (NFAT)-dependent IL-2 transcription, permitting alloreactive T cell activation (Phase 1) (5, 6). This triggers clonal expansion of T cells, which infiltrate the graft and target biliary epithelium and vascular endothelium (Phase 2) (7, 8), ultimately leading to biochemical and histological damage detectable by conventional methods (Phase 3) (9, 10). While this schema focuses on T cell–mediated rejection, antibody-mediated rejection can coexist and accelerate graft injury (11).

Current diagnostic approaches, including the Liver Graft Assessment Following Transplantation (L-GrAFT) score (12) and Model for Early Allograft Function Scoring (13), predominantly identify rejection only at Phase 3, when hepatocyte injury is already established. This diagnostic delay underscores a critical pharmacological gap in patient management. First, the reliance on late-phase biochemical markers means rejection is detected only after substantial inflammatory injury has occurred. Second, models fail to account for the fundamental drug-immune interaction, where the inflammatory state (e.g., CD4+ T cell activity) critically modulates the therapeutic efficacy of tacrolimus, as evidenced by the significant association between subtherapeutic tacrolimus levels and acute rejection (14). Third, while some models incorporate innate immunity markers or donor-specific antibodies, they neglect the core effector T cell populations that directly mediate the inflammatory response (15).

To bridge this pharmacological gap, we developed a streamlined immunopharmacologic model combining CD4+ T-cell percentage—an accessible marker of immune activation—with tacrolimus exposure. Rooted in the concept of conditional pharmacology, this approach seeks to identify high-risk patients exhibiting simultaneous immune activation and insufficient drug exposure. We propose that this strategy can open an early interventional window prior to the onset of irreversible inflammatory graft damage.

## Materials and methods

### Study design and population

This study aimed to develop a clinical decision-point risk stratification model for early graft dysfunction evaluation rather than an asymptomatic screening tool. This hypothesis-generating exploratory analysis was a single-center retrospective cohort study conducted at Beijing Chaoyang Hospital (August 2021-June 2024). For rejection cases, baseline measurements were intentionally defined at the first clinical suspicion of graft dysfunction to evaluate whether the model could provide superior acute rejection risk quantification using routinely available tests at this earliest point of clinical concern. The study aimed to identify potential immune signatures rather than test predefined hypotheses, thus no formal sample size calculation was performed *a priori*. The study protocol (IRB No. 2024-s-725) was approved by the Beijing Chaoyang Hospital Ethics Committee. Written informed consent was obtained for all peripheral-blood sampling; a waiver of consent was granted for retrospective collection of anonymized clinical data. All transplant procedures utilized organs from deceased Chinese citizens following post-mortem organ donation. The organ procurement and allocation strictly adhered to the ethical guidelines and laws of the People’s Republic of China.

The study population comprised liver transplant recipients managed at Beijing Chaoyang Hospital’s outpatient clinic. All patients were categorized into: (1) stable controls with stable liver function, and (2) rejection cases hospitalized for suspected acute rejection, confirmed by subsequent liver biopsy and reviewed by two independent hepatopathologists (Banff 2019 criteria) (16). The primary endpoint of this study was biopsy-proven ACR.

To emulate a prospective prediction scenario within our retrospective design, we employed a landmark analysis approach. For all patients, the ‘baseline’ measurements used in the model were defined as those obtained during routine clinical monitoring prior to any clinical suspicion of rejection. This ensures that the model predictions are based on information that would have been available to clinicians at the time of routine follow-up, thus validating its pre-emptive potential.

Exclusion criteria were systematically applied to eliminate confounding factors: (1) incomplete clinical or immunological data; (2) pre-existing immune-related conditions including autoimmune hepatitis, HIV infection, or hematologic malignancies; (3) ABO incompatible transplants; (4) re-transplantation or combined organ transplantation; (5) age < 18 years at the time of transplantation; (6) follow-up period < 6 months.

### Treatment protocol

All patients received intraoperative induction therapy with basiliximab (20 mg on day 0 and day 4 post-transplantation), as per our institutional standard protocol. The immunosuppressive regimen consisted of: (1) tacrolimus with initial target trough levels of 5-8 ng/mL, adjusted based on clinical response; (2) mycophenolate mofetil at 500 mg twice daily, with dose reduction to 250 mg twice daily for white blood cell counts 2.0-3.0×10^9/L and complete discontinuation for counts < 2.0×10^9/L; and (3) prednisone discontinued at 3 months post-transplant per institutional practice in patients with benign liver diseases, while switched to sirolimus at 1 month in those with kidney injury or with malignant indications (17–19).

For acute rejection episodes, tacrolimus dose adjustments followed a standardized protocol (20, 21): (1) primary escalation to target 8-11 ng/mL for rejection; (2) secondary augmentation with a round of steroids: intravenous methylprednisolone 500 mg on day 1, 240 mg on day 2, then daily reduction of 40 mg until 20 mg/day is reached, followed by oral prednisolone 20 mg/day for approximately one month if AST/ALT failed to decline > 50% within 72 hours or if neurotoxicity (tremor ≥ grade 2) occurred. Steroid-resistant cases (defined as < 50% improvement in liver biochemistry within 5 days) received rabbit anti-thymocyte globulin (Thymoglobulin, Genzyme) at 1.5 mg/kg/day for 5 days, preceded by rigorous screening for active infections.

### Flow cytometry analysis

Peripheral blood mononuclear cells (PBMCs) were analyzed following our established protocol (22, 23). The sequential gating strategy for lymphocyte subset identification is illustrated in **Supplementary Figure 1**. Briefly, PBMCs processed within 2 hours of collection to ensure > 95% viability (Trypan blue exclusion) were isolated from EDTA blood by Ficoll density centrifugation and stained with the following fluorochrome-conjugated antibodies from BD Biosciences: CD3-FITC (clone UCHT1, Cat. 555332), CD4-PerCP-Cy5.5 (clone SK3, Cat. 566923), CD8-PerCP-Cy5.5 (clone RPA-T8, Cat. 560662), CD19-PE (clone HIB19, Cat. 555413), CD56-PE (clone B159, Cat. 555516), CD16-APC (clone B73.1, Cat. 561304), CD11c-APC (clone B-ly6, Cat. 559877), CD123-PerCP-Cy5.5 (clone 7G3, Cat. 560904). Samples were acquired on a NovoCyte D2060R flow cytometer with a minimum of 10,000 viable lymphocytes per tube. Technical duplicates were run for 20% randomly selected samples to ensure reproducibility. Daily calibration was performed with CS&T beads, and compensation was verified using single-stained controls. Data were processed with NovoExpress software using standardized gating strategies.

### Definition of time points for model variables

Baseline tacrolimus levels and lymphocyte subset percentages were defined differently for the two study groups. In the non-rejection group, these values represent levels measured during routine outpatient monitoring, immediately prior to drug administration. In the rejection group, baseline referred to measurements obtained at the earliest indication of graft dysfunction, preceding the diagnostic liver biopsy. This included either the visit when liver function tests first demonstrated unexplained elevation, or the most recent routine monitoring visit before hospitalization for suspected rejection. Patients with biopsy-proven acute rejection underwent additional measurement approximately 2 weeks after the initiation of anti-rejection therapy.

### Statistical analysis

All statistical analyses were conducted using R software (version 4.3.1; R Foundation for Statistical Computing) with the following packages and methodologies: Normality of continuous variables was assessed using Shapiro-Wilk tests with visual confirmation by Q-Q plots. Between-group comparisons employed Student’s t-tests for normally distributed data or Mann-Whitney U tests for non-parametric distributions, while longitudinal analyses used paired t-tests or Wilcoxon signed-rank tests as appropriate. Multiple testing correction was performed using the Benjamini-Hochberg false discovery rate (FDR) method with α=0.05. Primary immunological endpoints were FDR-adjusted (q<0.05); all other comparisons are reported as nominal P values.

High-dimensional flow cytometry data were visualized using Uniform Manifold Approximation and Projection (UMAP; uwot package, version 0.1.16) with default parameters (n_neighbors=15, min_dist=0.1, metric=“euclidean”). Dynamic time warping (DTW) analysis was implemented through the dtw package (version 1.23-1) to quantify temporal patterns in lymphocyte subset changes, using the symmetric2 step pattern with open-end alignment. Euclidean metric was selected for DTW to preserve absolute magnitude differences in lymphocyte counts, as opposed to cosine similarity which would emphasize relative patterns. DTW was chosen over linear mixed models to capture non-linear temporal patterns in immunosuppression response. Distributional differences between groups were evaluated using Wasserstein distance metrics computed via the transport package (version 0.13-0). The Wasserstein distance quantifies the minimum work required to transform one probability distribution into another.

Given the small sample size and rare outcome (18 events among 98 patients), Firth-penalized logistic regression was employed to reduce small-sample bias and obtain more reliable estimates. Internal validation via 1000 bootstrap iterations provided optimism-corrected performance metrics. Multicollinearity among candidate predictors was assessed using variance inflation factors (VIFs) computed from linear models. VIF values < 5.0 were considered acceptable, indicating no substantial multicollinearity. CD3+ parameters were excluded from multivariate models due to collinearity with CD4+/CD8+ subsets (VIFs >5), as confirmed by the car package (version 3.1-2).

Model performance was assessed via the area under a receiver operating characteristic (AUC-ROC) analysis (pROC package, version 1.18.0), calibration curves, and decision curve analysis (rmda package, version 1.6), with 1,000 bootstrap iterations for internal validation. Sensitivity analyses with 20-fold multiple imputation confirmed robustness (ΔAUC <0.01). Lead-time analysis was performed to quantify the early warning capability of the model. For each patient who developed rejection and was identified as high-risk by the model, lead time was calculated as the interval between the date of model positivity (based on the Youden-optimized probability threshold) and the date of first biochemical evidence of graft injury (defined as ALT/AST > 2× upper limit of normal). The study is powered to detect Pearson correlations ≥ 0.30 (two-sided α = 0.05, power = 0.80) as calculated with the ‘pwr’ package; weaker associations may have been missed.

## Results

### Demographic and clinical characteristics

The baseline demographic and clinical characteristics of the study cohort are summarized in **Table 1**. Among the 98 liver transplant recipients, 18 developed biopsy-proven ACR (AR group) and 80 maintained stable graft function (Non-AR group). The median time to acute rejection was 5 months (range, 1-11 months). No statistically significant differences were observed in age, gender distribution, liver disease, MELD score, or key operative parameters including operating time, warm ischemia time, cold storage time, anhepatic phase duration, operative bleeding, and transfusion requirements between the two groups (all P > 0.05). Similarly, the distribution of immunosuppressive regimens or complications did not differ significantly between groups. This comparability strengthens the subsequent analysis by minimizing the confounding effects of these baseline variables.

**Table 1:** Comparison of characteristics between patients with and without acute rejection.

Variable	AR (n=18)	Non-AR (n=80)	P_Value
Gender (male)	16	62	0.3507
Disease(malignant)	8	17	0.069
Age	53.5 ± 8.78	50.19 ± 10.86	0.2304
MELD	14.38 (8.07-27.54)	14.62 (9.01-21.65)	0.8508
Operating time(min)	465 (392.5-537.5)	485 (420-562.5)	0.2125
Warm ischemia time(min)	1.5 (1-3)	2 (1-3)	0.1641
Cold storage time(min)	450 (375-480)	480 (417.5-480)	0.3371
Anhepatic phase(min)	65 (55-70)	70 (55-80)	0.3643
Operative bleeding(ml)	700 (400-800)	800 (500-1500)	0.1347
Transfusion(ml)	800 (800-1100)	1200 (550-2000)	0.3148
Complications			
Biliary Complications	1	7	1
Hemorrhage	3	9	0.6899
Infection	3	18	0.7556
Immunesuppression			0.2683
Tacrolimus+Sirolimus	6	16	
Tacrolimus	9	56	
Tacrolimus+MMF	3	8	

However, patients in AR group demonstrated significantly lower pre-treatment tacrolimus trough levels (5.15 ng/mL [3.85-6.30]) compared to non-rejection controls (6.60 ng/mL [4.90-7.80], p=0.0086). Following rejection therapy, tacrolimus levels increased to 8.25 ng/mL [7.15-9.35] (pre- vs post-treatment p<0.001). The rejection cohort showed higher peak ALT (498 U/L [145-820]) and bilirubin (3.4 mg/dL [2.1-5.0]) at diagnosis compared to stable controls (ALT < 60 U/L, bilirubin < 1.5 mg/dL). Post-treatment infection rates included cytomegalovirus viremia (11.1%), bacterial (22.2%), and fungal infections (16.7%).

### Lymphocyte subset profiles in stable liver transplant recipients

**Figure 1:**
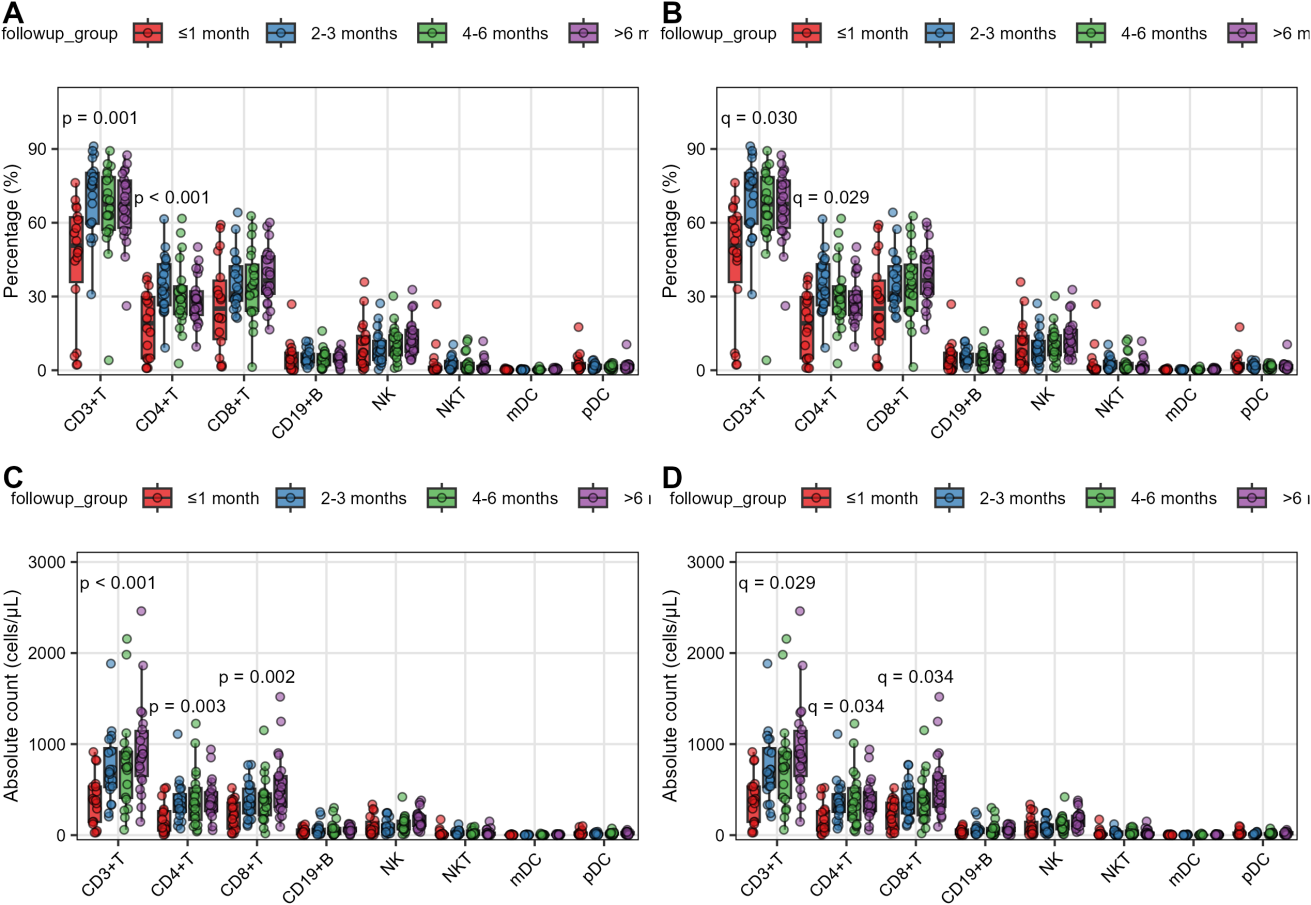
Temporal immune reconstitution in stable liver transplant recipients. **(A, B)** Percentages and **(C, D)** absolute counts of major lymphocyte subsets across four post-transplant intervals. Data are presented as box plots showing median (central line), interquartile range (box), and 10th-90th percentiles (whiskers). Individual data points are overlaid. Statistical comparisons were performed using Kruskal-Wallis test **(A, C)** with Benjamini-Hochberg false discovery rate (FDR) correction **(B, D)** for multiple comparisons. Time points: ≤1 month (n=18), 2-3 months (n=20), 4-6 months (n=20), >6 months (n=22) post-transplant.

Comparative analysis revealed significant temporal changes in T cell subsets during post-transplant follow-up (FDR-adjusted q < 0.05, **Supplementary Table 1**; **Figure 1**): CD3+T% increased from 44.7 ± 24.5% at ≤ 1 month to 66.7 ± 14.4% at > 6 months (p < 0.001, q = 0.029), while CD4+T% showed early elevation from 18.3 ± 13.4% to 34.8 ± 11.9% at 2-3 months (p < 0.001, q = 0.029). Absolute counts demonstrated parallel increases, with CD3+T cells rising from 388 ± 278 to 951 ± 510 cells/μL (p < 0.001, q = 0.029), CD4+T cells from 170 ± 171 to 404 ± 213 cells/μL (p < 0.001, q = 0.034), and CD8+T cells exhibiting delayed recovery from 220 ± 158 to 549 ± 344 cells/μL (p < 0.001, q = 0.034). No significant differences were observed in any lymphocyte subsets when stratified by sex (male vs female, all q > 0.73), age groups (≥ 60 vs < 60 years, all q > 0.91), or primary disease (malignant vs benign, all q > 0.62) (**Supplementary Figures 2**-**4**).

UMAP analysis identified strong correlation between UMAP1 coordinates and CD4+T cell parameters (CD4+T%: r = 0.49, p < 0.001; CD4+T count: r = 0.58, p < 0.001). UMAP2 primarily reflected CD8+T cell distribution (CD8+T%: r = 0.69, p < 0.001). Early post-transplant patients (≤ 1 month) clustered distinctly in UMAP space (Wasserstein distance=3.21, **Supplementary Figure 5**). The immune landscape remains relatively homogeneous across sex, age, and primary liver disease in stable transplant recipients (**Supplementary Figure 6**).

These findings suggest distinct temporal reconstitution patterns for major T cell populations, while other lymphocyte subsets remained stable across demographic and etiological subgroups.

### Dynamic changes in lymphocyte subsets before and after anti-rejection therapy

**Figure 2:**
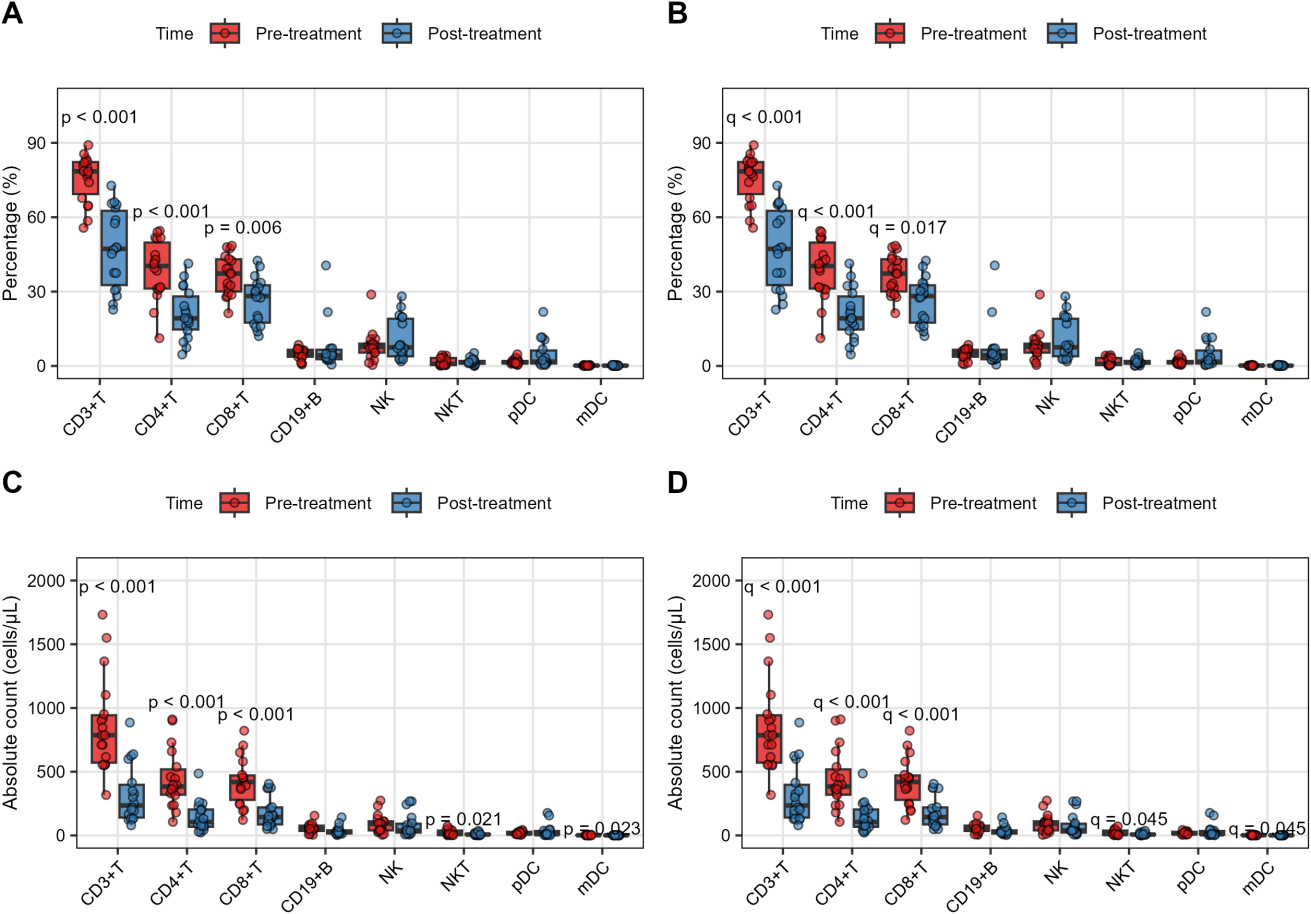
Dynamic changes in lymphocyte subsets following anti-rejection therapy. **(A, B)** Percentages and **(C, D)** absolute counts of lymphocyte subsets before (red) and after (blue) anti-rejection treatment in 18 patients with biopsy-proven acute rejection. All data points are fully paired (same patients pre- and post-treatment). Statistical comparisons were performed using Wilcoxon signed-rank test **(A, C)** for non-normally distributed data with Benjamini-Hochberg FDR correction **(B, D)**. The results demonstrate selective depletion of T-cell subsets, particularly CD4+ and CD8+ T cells, following therapy.

To evaluate the immunological alterations following anti-rejection treatment in liver transplant recipients, we compared peripheral blood lymphocyte subsets (both percentages and absolute counts) in 18 patients before and after therapy. CD3+T cells decreased from 75.67 ± 9.53% to 47.20 ± 16.05% (p < 0.001, q < 0.001); CD4+T cells declined from 38.82 ± 11.97% to 20.96 ± 10.18% (p < 0.001, q < 0.001). CD8+T cells showed moderate reduction (36.65 ± 8.00% vs. 26.36 ± 9.27%, p < 0.001, q < 0.001). No significant changes occurred in CD19+B cells, NK cells, NKT cells, or dendritic cell subsets (all q > 0.05) (**Figures 2A, B**; **Supplementary Table 2**).

More dramatic depletion was observed in absolute counts. CD3+T cells plummeted from 861 ± 372 to 320 ± 227 cells/μL (p < 0.001, q < 0.001). CD4+T cells dropped from 441 ± 228 to 143 ± 116 cells/μL (p < 0.001, q < 0.001). CD8+T cells decreased from 420 ± 186 to 178 ± 122 cells/μL (p < 0.001, q < 0.001). NKT cells showed marginal reduction (20.7 ± 21.4 vs. 10.3 ± 9.9 cells/μL, p = 0.021, q = 0.045). CD19+B cells and NK cells demonstrated non-significant downward trends, while dendritic cells remained stable (all q > 0.05) (**Figures 2C, D**; **Supplementary Table 2**). This synchronous T-cell depletion likely reflects glucocorticoid receptor-mediated apoptosis (24), while preserved innate immunity suggests calcineurin inhibitor-specific effects on nuclear factor of activated T-cells signaling (25).

The data reveal selective depletion of T lymphocyte subsets, particularly CD4+T and CD8+T populations, with relative preservation of innate immune cells after anti-rejection therapy. The magnitude of reduction was more pronounced in absolute counts than percentages, suggesting both proportional redistribution and genuine cell loss.

### Longitudinal immune dynamics after anti-rejection therapy

**Figure 3:**
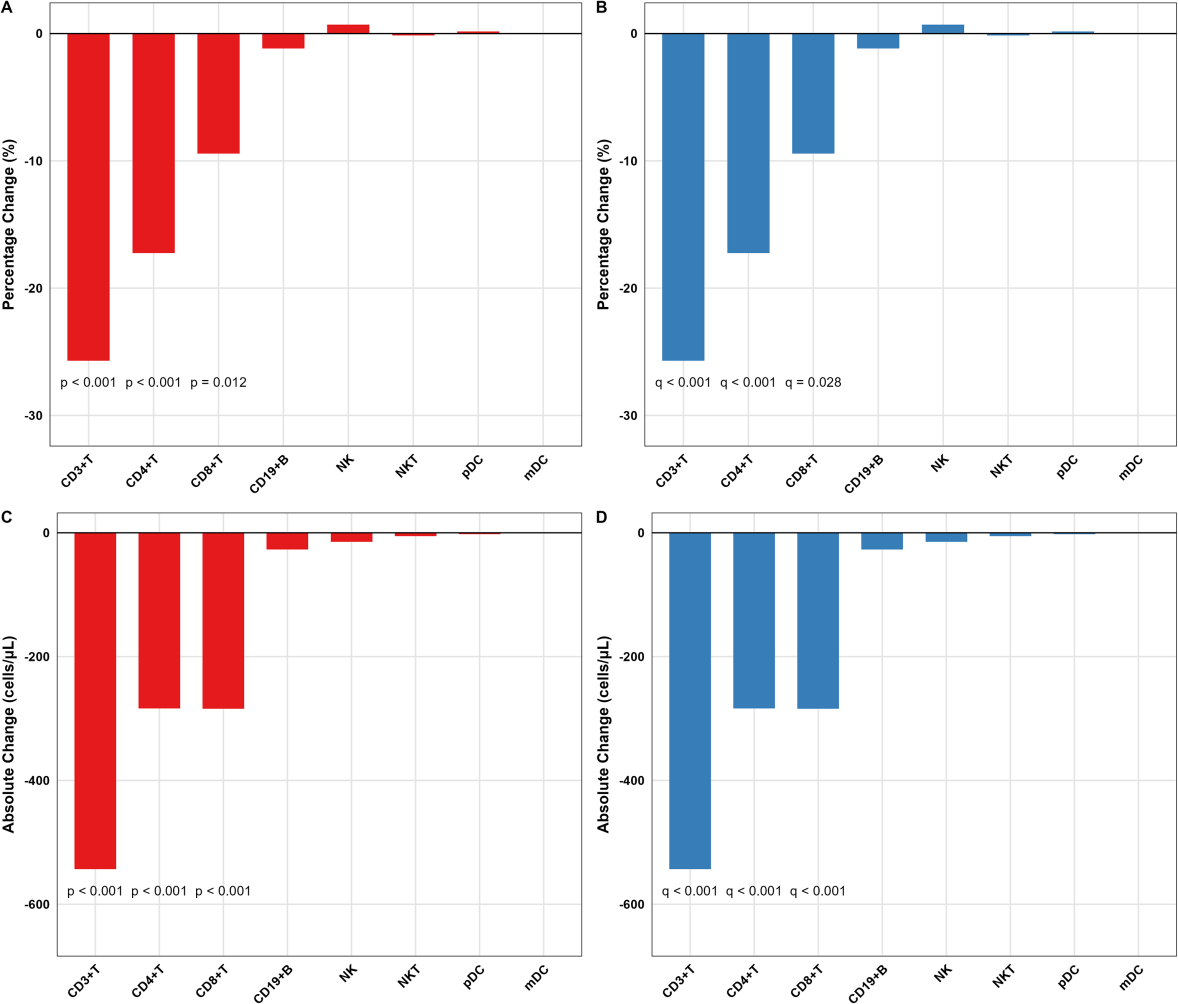
Magnitude of change in lymphocyte subsets after anti-rejection therapy. **(A, B)** Changes in percentages and **(C, D)** absolute counts (Δ = post-treatment minus pre-treatment) for each lymphocyte subset. Negative values indicate reduction after therapy. Statistical significance was assessed using one-sample Wilcoxon signed-rank test **(A, C)** against zero with Benjamini-Hochberg FDR correction **(B, D)**. The data confirm the significant reduction in T-cell compartments as the primary immunological response to anti-rejection therapy.

Anti-rejection therapy triggered coordinated declines in both the percentage and absolute counts of major T cell compartments (**Figure 3**; **Supplementary Table 3**). Specifically, CD3+T cell percentage dropped by a median of 25.7% (q < 0.001), while CD3+T, CD4+T and CD8+T absolute counts declined by 543, 284 and 284 cells/μL, respectively (all q < 0.001). A parallel fall in CD4+T cell percentage (median −17.2%, q < 0.001) and CD8+T cell percentage (median −9.4%, q = 0.028) was observed. Among non-T subsets, only myeloid dendritic cells (mDC) and NKT cells exhibited modest absolute reductions (median −0.9 cells/μL and −10.4 cells/μL; p = 0.023 and 0.021, respectively), although these did not withstand FDR correction. CD19+B cell and NK cell parameters remained stable (q > 0.05). Spearman correlation analysis of the significant change scores (Δ) demonstrated an inverse relationship between ΔCD4+T cell percentage and ΔCD8+T cell percentage (r = −0.43, p = 0.046) and a positive correlation between ΔCD4+T cell percentage and ΔCD4+T cell absolute change (r = 0.59, p = 0.012), indicating coupled CD4 dynamics during immune recovery (**Supplementary Figures 7, 8**).

Then, to quantify the temporal coherence of these changes, DTW analysis revealed pre- and post-treatment time-series of all 16 lymphocyte parameters. DTW distance ranking highlighted CD3+T cell absolute count, CD4+T cell absolute count, and CD8+T cell absolute count as the three most consistently reshaped subsets (mean DTW distances 28.5, 17.9 and 10.3, respectively), whereas CD19+B-cell, NK, pDC and NKT compartments showed low distances, underscoring minimal or inconsistent responses (**Supplementary Figure 9**; **Supplementary Table 4**). Heat-map visualization confirmed a uniform red-to-blue shift (decrease) across the T-cell zone, while non-T subsets remained color-neutral (**Supplementary Figure 10**). Trajectory plots further revealed tight convergence of individual patient lines toward lower T cell values (**Supplementary Figure 11**), contrasting with the widely scattered paths of B and NK cells (**Supplementary Figure 12**).

Taken together, DTW analysis corroborates the selective and synchronous suppression of T-cell subsets as the hallmark of effective anti-rejection therapy.

### Distinct lymphocyte profiles in liver transplant recipients with and without acute rejection

**Figure 4:**
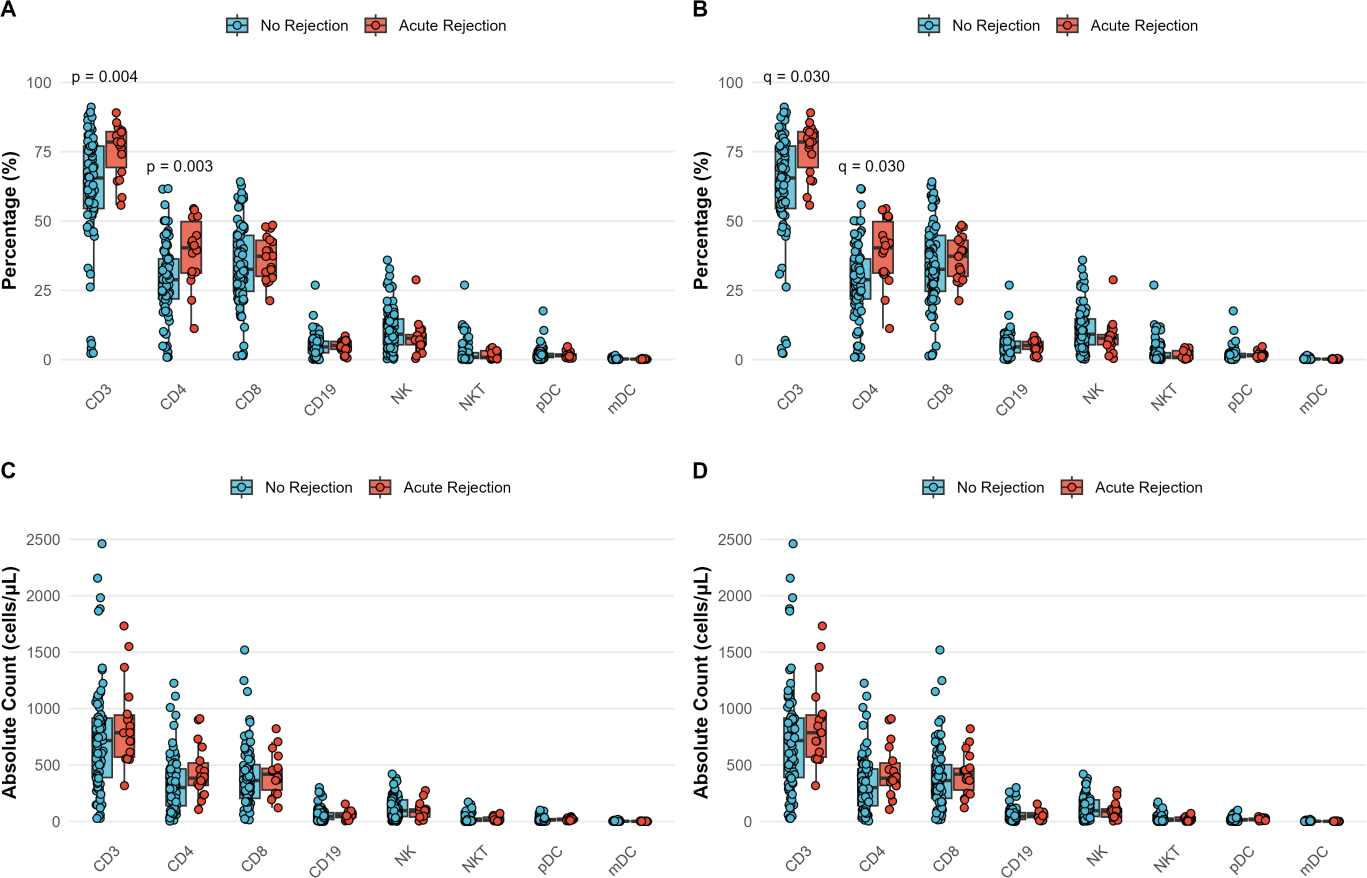
Differential lymphocyte profiles between non-rejection and acute rejection patients. **(A, B)** Percentages and **(C, D)** absolute counts of lymphocyte subsets in stable transplant recipients (n=80, blue) versus patients with biopsy-proven acute rejection (n=18, red). Statistical comparisons were performed using Mann-Whitney U test **(A, C)** for non-normally distributed data with Benjamini-Hochberg FDR correction **(B, D)**. The data show significantly elevated CD4+ T-cell percentages in rejection patients, while other subsets remain comparable.

Comparative analysis revealed significant differences in lymphocyte subsets between non-rejection (n=80) and pre-treatment acute rejection (n=18) groups (**Figure 4**; **Supplementary Table 5**). CD3+T cell and CD4+ T cell percentages were markedly higher in rejection patients (CD3+T: 78.5% [69.3-82.3] vs 65.5% [54.5-77.0], q = 0.030; CD4+T:38.8 ± 12.0% vs 28.5 ± 13.4%, q = 0.030). The volcano plot highlighted CD3+% and CD4+% as the most differentially distributed subsets (effect sizes >1.2, **Supplementary Figure 13A**), confirmed by boxplot visualization (**Supplementary Figure 13B**). No significant differences were observed in CD8+T cells, NK/NKT cells, or DC subsets (all q > 0.05). Clinical parameters (age, sex, follow-up duration) and primary liver disease distribution were comparable between groups (**Supplementary Figure 14**).

This integrated analysis demonstrates that acute rejection is associated with specific alterations in T cell homeostasis, while innate immunity remains preserved. The combination of traditional statistics with distribution-based metrics (Wasserstein distance) provides robust characterization of rejection-associated immune signatures.

### Immunopharmacologic signature for acute rejection prediction

**Figure 5:**
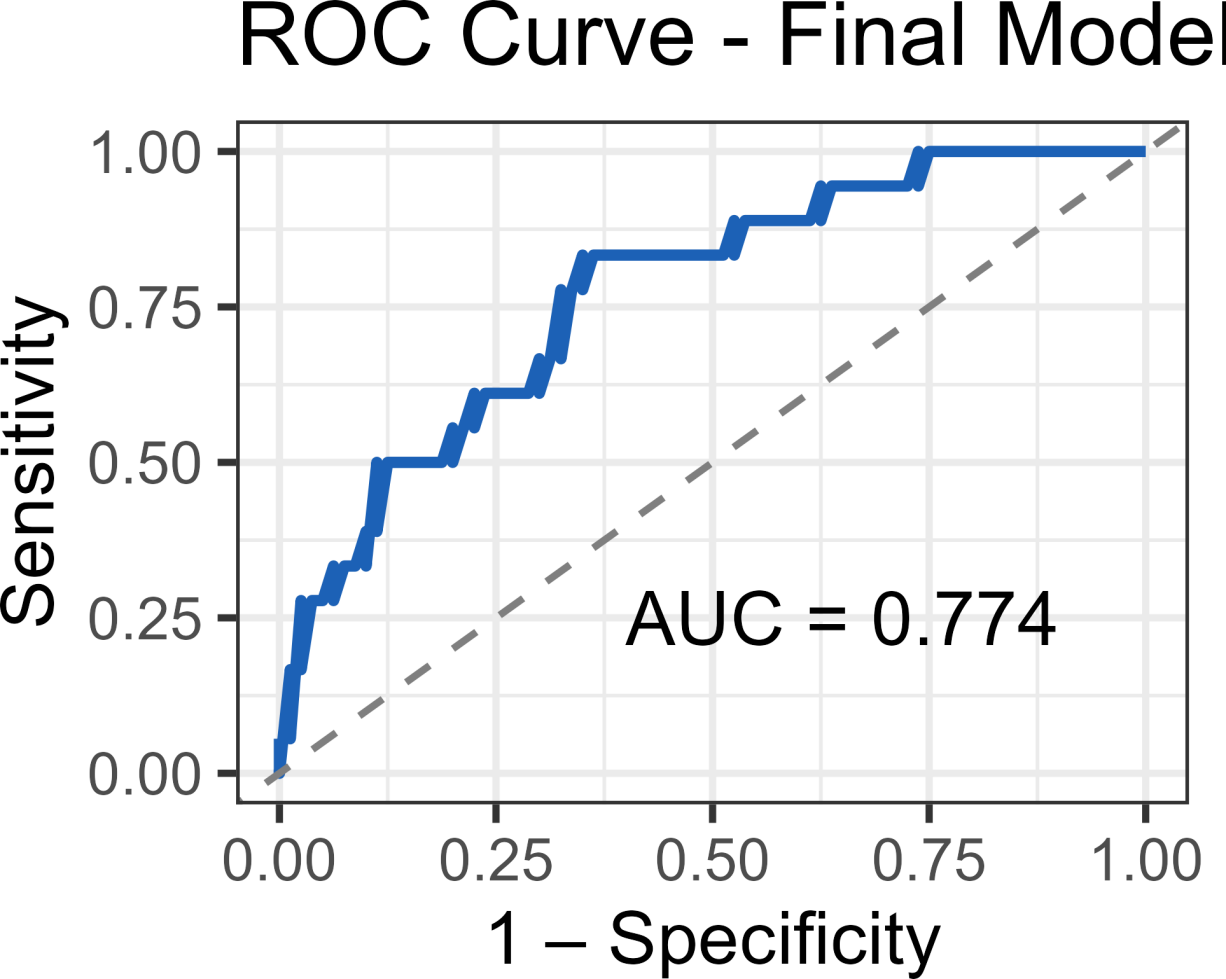
Receiver operating characteristic (ROC) curve of the immunopharmacologic prediction model. The ROC curve illustrates the diagnostic performance of the final model combining tacrolimus trough level and CD4+ T-cell percentage for predicting acute rejection. The area under the curve (AUC) is 0.774 (95% CI: 0.674-0.874). The dashed diagonal line represents chance performance (AUC = 0.5). The optimal cut-point identified by Youden’s index provides 66.7% sensitivity and 83.8% specificity.

Guided by univariate analysis identifying tacrolimus levels (OR 0.739, 95% CI 0.555-0.935, p=0.009) and CD4+ T cell percentage (OR 1.060, 95% CI 1.020-1.110, p=0.004) as significant correlates of acute rejection, we constructed a parsimonious prediction model. Comprehensive evaluation of other lymphocyte subsets revealed no additional independent predictive value beyond the core CD4+ T cell-tacrolimus axis. In 98 recipients (18 rejection, 80 non-rejection), the final Firth-penalized logistic model retained only baseline tacrolimus (OR per ng/mL 0.732, 95% CI 0.537-0.947, p=0.015) and baseline CD4+ T cell percentage (OR per % 1.072, 95% CI 1.020-1.137, p=0.005) as independent predictors (**Supplementary Figure 15**). Collinearity diagnostics confirmed statistical independence between the predictors, with variance inflation factors of 1.04 for both tacrolimus level and CD4+ T-cell percentage, and a weak negative correlation (r = -0.205, P = 0.415). The model demonstrated good discrimination with an AUC of 0.774 (95% CI 0.674-0.874; **Figure 5**), sensitivity 66.7%, specificity 83.8%, and a Brier score of 0.123. Bootstrap internal validation (R = 1,000) confirmed model stability with a median optimism-corrected AUC of 0.785 (95% CI 0.685-0.885). The model exhibited excellent calibration across the risk spectrum (Hosmer-Lemeshow test: χ²=3.166, p=0.924; **Supplementary Figure 16**), and decision curve analysis demonstrated net clinical benefit across threshold probabilities of 10-50% (**Supplementary Figure 17**).

For clinical implementation, risk stratification using the optimal probability threshold of 0.178 provided 83.3% sensitivity and 65.0% specificity. Patients with both high CD4+ T% (≥30.1%) and low tacrolimus levels (<6.2 ng/mL) had substantially elevated rejection risk (Fisher’s exact p<0.001), identifying 72.2% (13/18) of rejection cases while maintaining 85.0% specificity.

**Figure 6:**
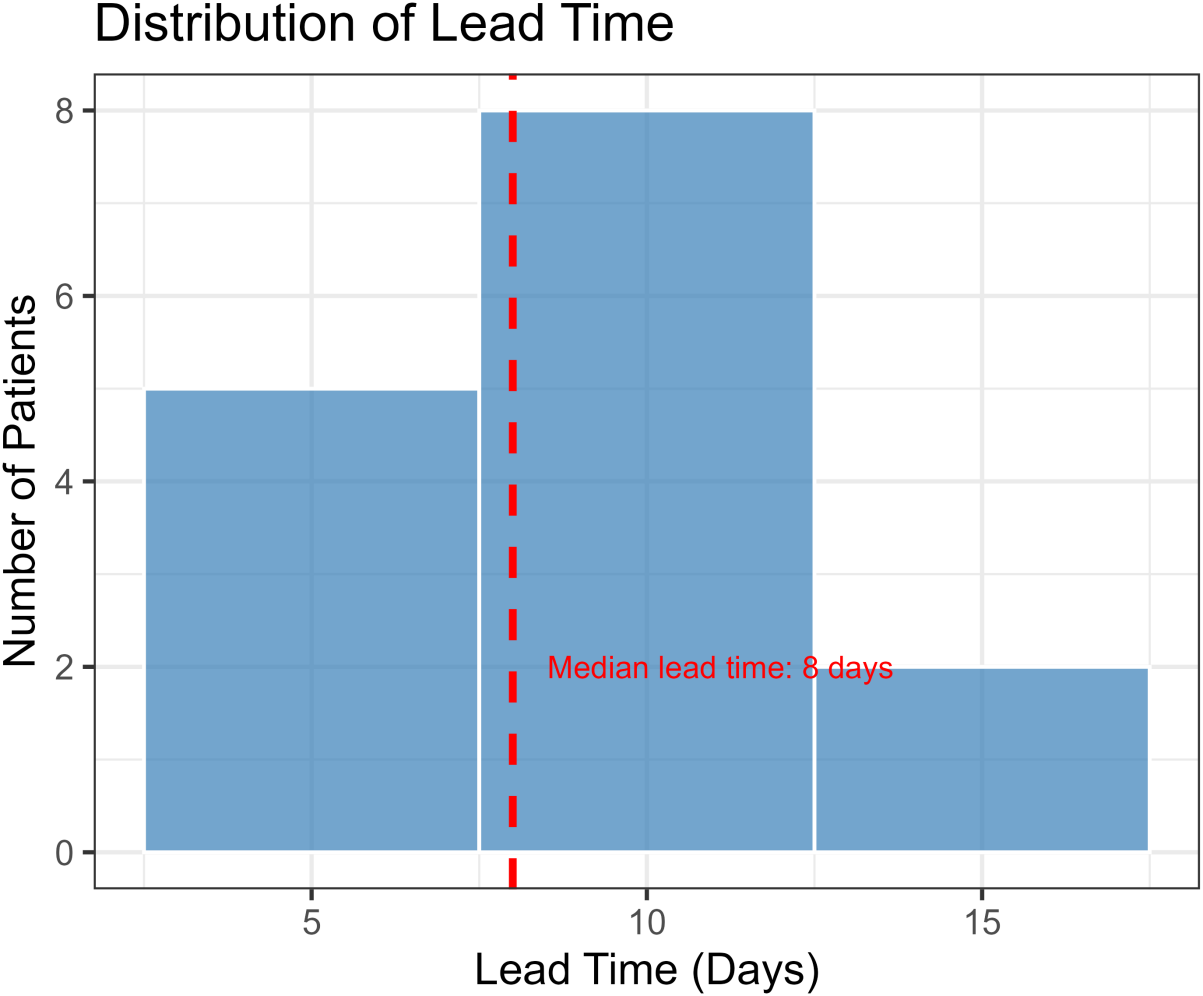
Lead time analysis of the immunopharmacologic prediction model. Swimlane plot showing the temporal relationship between model positivity, biochemical evidence of graft injury, and clinical diagnosis in patients who developed acute rejection. Each horizontal line represents one patient (n=15 model-positive patients). The median lead time between model positivity and biochemical injury was 8 days (IQR: 3.5 days; range: 6-14 days). All model-positive patients showed positive lead times, demonstrating consistent early detection capability.

Critically, lead-time analysis was performed on patients identified as high-risk by the model using the Youden’s index-optimized threshold (probability > 0.178), which corresponded to 15 model-positive patients (83.3% of the rejection cohort), providing early warning signals a median of 8 days (IQR: 3.5 days; range: 6-14 days) before biochemical evidence of graft injury emerged (**Figure 6**). Among these model-positive patients, all (15/15, 100%) showed positive lead times, confirming consistent early detection when the model identified rejection risk.

The combined model provided significant incremental value over individual components (**Supplementary Figure 18**), with absolute AUC improvements of 0.080 over tacrolimus monitoring alone (0.774 vs. 0.694) and 0.041 over CD4+ T cell monitoring alone (0.774 vs. 0.733). Likelihood ratio tests confirmed these improvements were statistically significant (p = 0.007 and p = 0.014, respectively).

Collectively, this streamlined two-variable immunopharmacologic model enables pre-emptive risk stratification for acute liver-transplant rejection, offering both early detection capability and clinically meaningful performance improvements over conventional monitoring approaches.

## Discussion

Our study establishes a practical immunopharmacologic framework for preemptively predicting ACR by integrating CD4+ T cell profiling with tacrolimus pharmacodynamics. Critically, our lead-time analysis provides direct evidence of genuine pre-emptive capability, demonstrating a median 8-day warning window before biochemical injury emerges. The lead-time analysis demonstrates the model’s potential to advance the detection timeline from the point of established biochemical injury to earlier risk identification, which could enable preventive interventions before significant graft damage occurs. This model directly addresses the clinical need to identify rejection risk in the pre-injury phase, filling a gap left by current approaches that detect dysfunction only at later stages.

Our model is positioned as a clinical decision-support tool for the early phase of graft dysfunction evaluation, not as a universal screening instrument. The primary application scenario is risk stratification of liver transplant recipients presenting with early, mild, or non-specific liver function abnormalities. A high-risk result (elevated CD4+ percentage combined with subtherapeutic tacrolimus) should strongly suggest acute rejection, prompting consideration of tacrolimus dose optimization or early biopsy. Conversely, a low-risk result supports exploring alternative etiologies (e.g., infection, drug-induced liver injury), potentially avoiding unnecessary invasive procedures or excessive immunosuppression. Such risk-based stratification represents a crucial step toward personalized treatment. The ‘asymmetric’ timepoint definition in our study reflects this intended use case: providing risk stratification when patients first present with concerning but non-diagnostic findings. This pragmatic approach addresses a critical gap in current management, where clinicians lack tools to distinguish immune-mediated rejection from other causes of early graft dysfunction. Furthermore, comprehensive model comparisons confirm significant incremental value, with statistically meaningful AUC improvements over single-parameter monitoring (11.4% vs. tacrolimus alone, p=0.007; 5.5% vs. CD4+ T cells alone, p=0.014). This demonstrates that the combination provides clinically important enhancements beyond conventional monitoring.

When contextualized within the current landscape of rejection prediction, our model’s performance (AUC 0.774) compares favorably with other rejection-specific prediction tools. The recent multicenter model by Chen et al (26) achieved AUCs of 0.713 in adults and 0.786 in children using clinical and laboratory parameters, while our immunologically-focused approach provides comparable discrimination with greater biological specificity. More importantly, our model offers practical advantages through its reliance on routinely available flow cytometry and therapeutic drug monitoring, avoiding the complexity of genomic profiling required by approaches like the 59-gene classifier (AUC 0.83) described by Levitsky et al (27). It is important to distinguish our rejection-specific model from broader early allograft dysfunction (EAD) prediction scores. The well-validated L-GrAFT7 risk score, which demonstrates excellent performance in predicting 3-month graft failure (AUROC 0.78-0.85 across multiple cohorts) (12, 28), serves a different clinical purpose by assessing overall graft function rather than specifically identifying immune-mediated rejection.

The distinctive value of our immunopharmacologic signature lies in its ability to capture the dynamic interplay between immunosuppression exposure and immune activation as our findings are strongly supported by the established role of CD4+ T cells in rejection pathogenesis. The observed elevation in CD4+ T cell percentages among rejection patients aligns with the recognized sequence of allorecognition, where CD4+ T cells initiate the rejection cascade through indirect antigen presentation and subsequent cytokine-driven amplification (29). The selective depletion of CD4+ T cells following anti-rejection therapy further validates the biological relevance of our monitoring target. The clinical implementation of CD4+ T-cell percentage monitoring requires consideration of several practical factors. While this parameter can be influenced by concurrent conditions (e.g., infections, corticosteroid bursts) and sampling timing, its integration with tacrolimus levels in our model provides contextual interpretation. An elevated CD4+ percentage is primarily concerning when accompanied by subtherapeutic tacrolimus exposure. Standardization of sampling protocols (e.g., pre-dose timing) and integration with infection screening would enhance reproducibility in clinical practice.

The integration of tacrolimus levels provides crucial pharmacodynamic context, as subtherapeutic exposure permits NFAT-dependent IL-2 transcription and subsequent T cell proliferation (30). This combination of immune activation and inadequate suppression creates the high-risk phenotype our model identifies, offering a more nuanced assessment than either parameter alone.

In the evolving landscape of transplant monitoring, donor-derived cell-free DNA (dd-cfDNA) has emerged as a promising biomarker. Studies by Levitsky et al (31) have demonstrated impressive performance (AUC 0.95 for rejection vs normal function) and the ability to detect rejection up to 100 days before clinical manifestation. While promising, dd-cfDNA detects established graft injury rather than impending risk, potentially limiting preemptive utility. In contrast, our model provides both risk stratification and actionable mechanistic insights—directly informing tacrolimus escalation decisions. Beyond cellular and genomic approaches, predictive models based on soluble immune mediators or circulating miRNAs have shown promise in specific contexts (32, 33). While these methods offer different biological insights, our model’s reliance on routine clinical tests (flow cytometry and therapeutic drug monitoring) provides distinct advantages in immediate clinical applicability, cost-effectiveness, and integration into existing workflows. The choice among these complementary approaches should consider the specific clinical context and available resources. We propose a combined monitoring strategy: immunophenotyping for routine screening and dd-cfDNA for confirmatory testing. This approach leverages dd-cfDNA’s sensitivity while retaining our model’s therapeutic guidance. Clinically, identifying high-risk patients (elevated CD4+% + subtherapeutic tacrolimus) enables preemptive intervention, while low-risk profiles may permit reduced monitoring intensity.

We acknowledge several limitations inherent to this exploratory study. First, the model was derived from a single-center cohort with a limited number of rejection events (n=18). Although we employed statistical techniques specifically designed for such settings (Firth regression, bootstrapping) and observed stable performance in internal validation, external validation in larger, multicenter prospective cohorts is essential to confirm generalizability. Second, the model is based on a single time-point assessment, which may not fully capture dynamic immune-pharmacologic interactions; future studies employing serial monitoring could enhance sensitivity. Third, as a risk stratification tool developed using data from the ‘point of clinical suspicion,’ our model is not directly equivalent to a universal screening tool for completely healthy, asymptomatic transplant recipients. Future prospective validation at fixed, asymptomatic timepoints is needed to explore its potential as a broader pre-emptive prediction tool for routine monitoring. The differing baseline definitions—routine monitoring for stable patients versus the onset of clinical suspicion for rejection cases—reflects this pragmatic design but could theoretically inflate discriminatory performance. A prospective study with uniform timepoints is warranted to provide an unbiased performance estimate. Fourth, the absence of pre-transplant immune profiles limits our ability to contextualize post-transplant changes relative to individual baselines. Future prospective studies should incorporate pre-transplant immunophenotyping to establish personalized reference ranges. Fifth, the cohort primarily comprised recipients with viral hepatitis or HCC under a specific immunosuppressive protocol (including sirolimus conversion), which may reflect regional practices. The validation of this model across diverse etiologies, ethnicities, and immunosuppressive regimens is essential. Sixth, while our model focuses on the critical effector arm (CD4+ T cells) and its pharmacological control, we acknowledge the potential role of regulatory immune subsets in transplant immunology. Specialized populations such as CD56bright NK cells, which require specific markers (CD127, CD45) for identification (34), were not measured in our retrospective study utilizing routine clinical flow cytometry panels. Future prospective studies incorporating comprehensive immunophenotyping could evaluate whether inclusion of these regulatory subsets further refines risk stratification approaches. Finally, unmeasured confounders such as CYP3A5 genotype and medication adherence may influence the tacrolimus-CD4+ T cell relationship, partly explaining why not all patients with subtherapeutic exposure develop rejection.

## Conclusions

In conclusion, our study provides a biologically grounded and clinically actionable tool for ACR risk stratification. By leveraging routinely available parameters, it presents a feasible pathway towards personalized and preemptive immunosuppression management, potentially transforming patient outcomes after liver transplantation.

## Data Availability

The raw data supporting the conclusions of this article will be made available by the corresponding authors, without undue reservation.
