# Supplementary material for: A pre-emptive risk model for acute rejection in liver transplantation: an immunopharmacologic biomarker panel combining CD4+ T-cell profiling and tacrolimus exposure
Source: Front Immunol. 2026 Mar 3;17:1760409. doi: 10.3389/fimmu.2026.1760409 (PMC12992057; doi:10.3389/fimmu.2026.1760409)
Supplement: Supplementary file 6 [file DataSheet6.pdf]

Supplementary information for

A Pre-emptive Risk Model for Acute Rejection in Liver Transplantation: An Immunopharmacologic Biomarker Panel Combining CD4+ T-Cell Profiling and Tacrolimus Exposure

Qin-Xin Li[a*], M.M, liqinxin8641@163.com; Jun-Xi Zhang[a*], M.M, 17629963625@163.com; Han Li[b]*, lihanw11@163.com；Xian-Liang Li[a], M.D., lixianliangbjcy@126.com; Qiang He[a], M.D., heqiang349@sina.com; Dong-Dong Han[c#], surghandd@163.com; Ji-Qiao Zhu[a#], M.D., dr_zhujiqiao@163.com

[a]Department of Hepatobiliary and Pancreaticosplenic Surgery, Medical Research Center, Beijing Organ Transplant Center, Beijing Chaoyang Hospital, Capital Medical University, No.8 Gongtinan Road, Chaoyang District, Beijing 100020, China

[b]Department of Head and Neck Surgery, National Cancer Center/National Clinical Research Center for Cancer/Cancer Hospital, Chinese Academy of Medical Sciences and Peking Union Medical College Beijing 100021, China

[c]Department of Hepatobiliary Surgery, China-Japan Friendship Hospital, Beijing 100029, China
*These authors contributed equally to this work

[#]Corresponding authors: Ji-Qiao Zhu and Dong-Dong Han

Dong-Dong Han, No. 2 Yinghua East Street, Chaoyang District, Beijing 100029, PR China, Phone +86-(0)-10-84205528, Fax+86-(0)-10-84205528

Ji-Qiao Zhu, No. 8 Gongti South Road, Chaoyang District, Beijing 100020, PR China, Phone +86-(0)-10-85231504, Fax +86-(0)-10-85231503

Supplementary figures and figure legends

Supplementary Figure S1. Gating strategy for flow cytometry analysis.

Sequential gating strategy for identification of peripheral blood lymphocyte subsets: (i) Forward scatter-area (FSC-A) vs side scatter-area (SSC-A) to identify cells, (ii) FSC-A vs FSC-height (FSC-H) to exclude doublets, (iii) viability staining to select live cells, (iv) CD3+ for T cells with subsequent CD4+ and CD8+ gating, (v) CD3-CD19+ for B cells, (vi) CD3-CD56+/CD16+ for NK cells, (vii) CD3+CD56+ for NKT cells, and (viii) CD11c+/CD123+ for dendritic cell subsets (mDC: CD11c+CD123-; pDC: CD11c-CD123+).

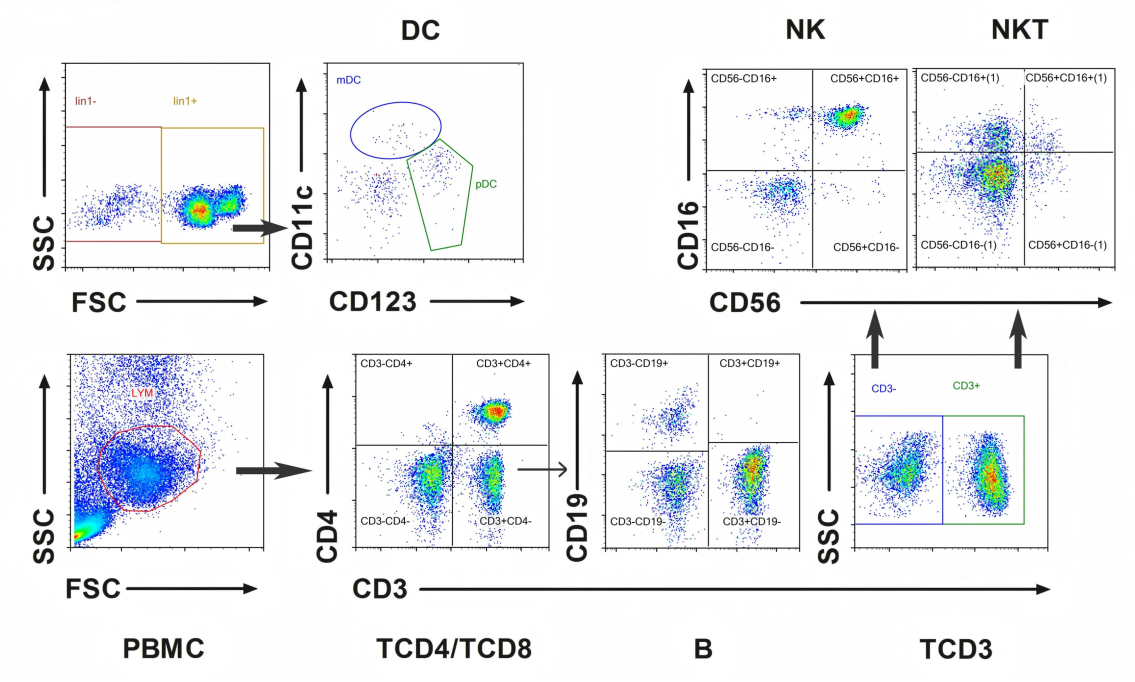

Supplementary Figure S2. Lymphocyte subset distribution stratified by age

(A,B) Percentages and (C,D) absolute counts of lymphocyte subsets in stable transplant recipients stratified by age (<60 years, n=66 vs ≥60 years, n=14). Statistical comparisons were performed using Mann-Whitney U test (A,C) with Benjamini-Hochberg FDR correction (B,D). No significant differences were observed across any lymphocyte subsets between age groups.

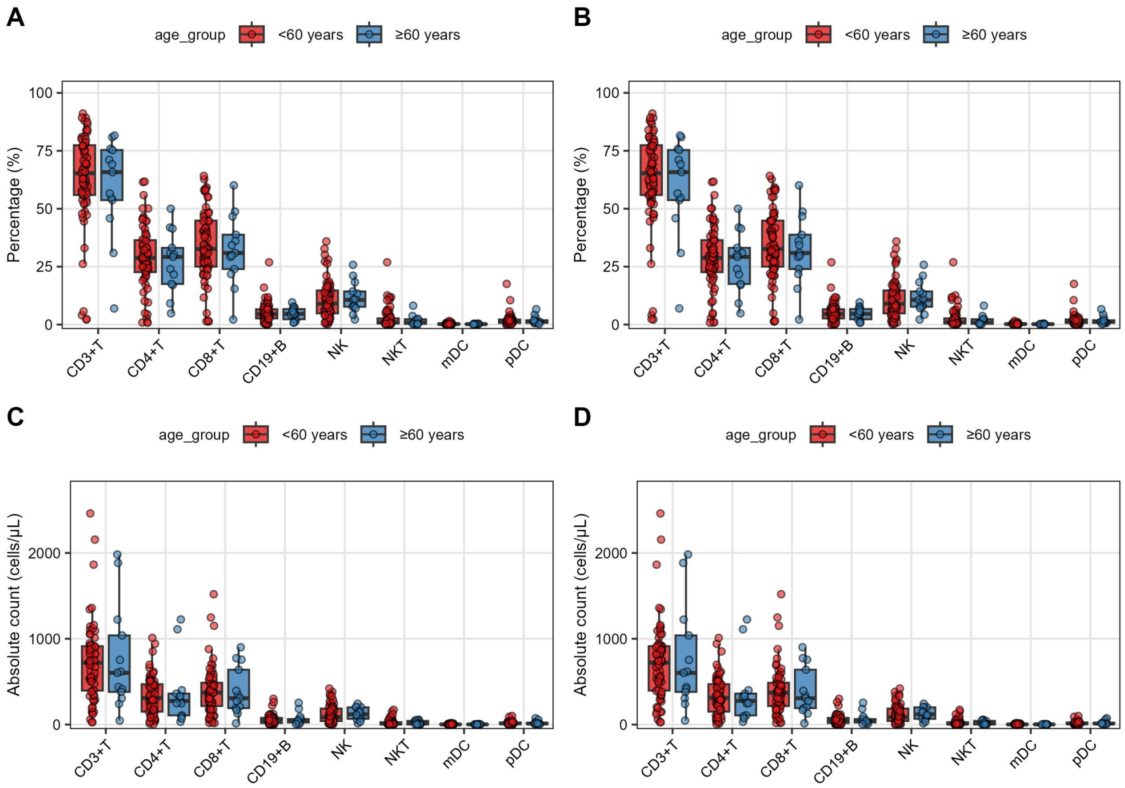

Supplementary Figure S3. Lymphocyte subset distribution stratified by primary liver disease.

(A,B) Percentages and (C,D) absolute counts of lymphocyte subsets in stable transplant recipients with benign (n=45) versus malignant (n=35) liver diseases. Statistical comparisons were performed using Mann-Whitney U test (A,C) with Benjamini-Hochberg FDR correction (B,D). No significant differences were observed across any lymphocyte subsets between disease categories.

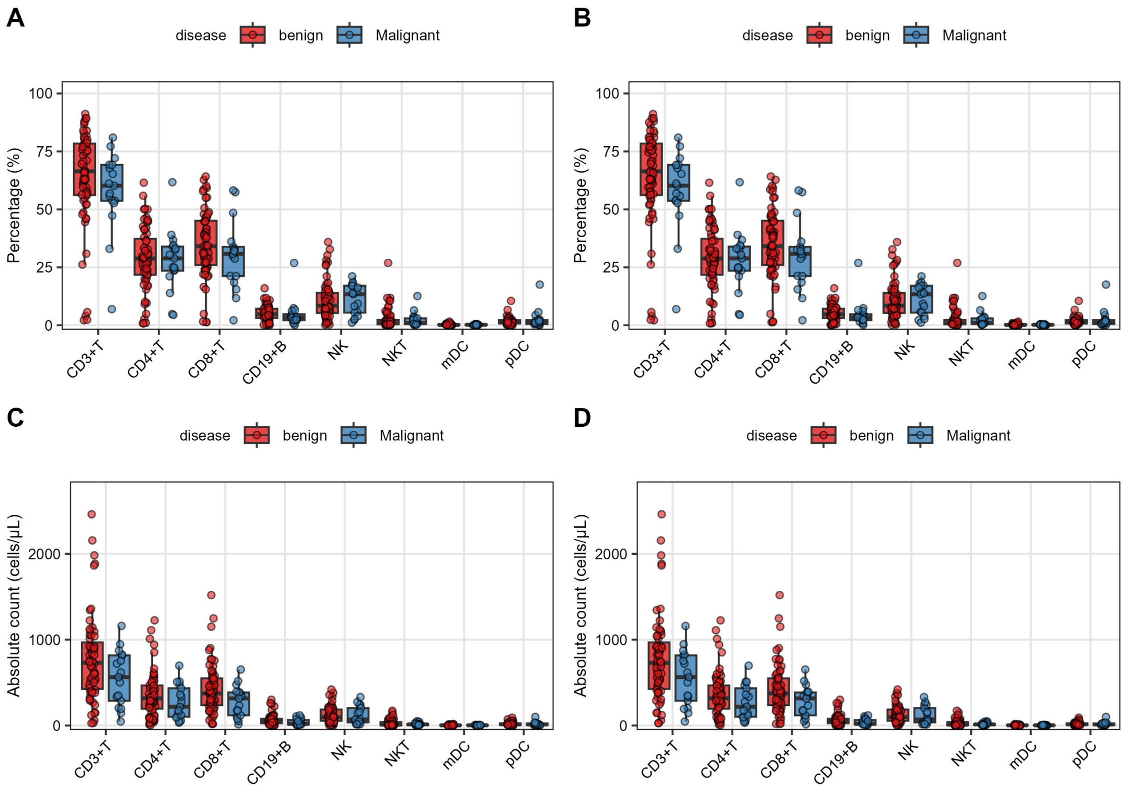

Supplementary Figure S4. Lymphocyte subset distribution stratified by sex

(A,B) Percentages and (C,D) absolute counts of lymphocyte subsets in stable male (n=62) versus female (n=18) transplant recipients. Statistical comparisons were performed using Mann-Whitney U test (A,C) with Benjamini-Hochberg FDR correction (B,D). No significant differences were observed across any lymphocyte subsets between sexes.

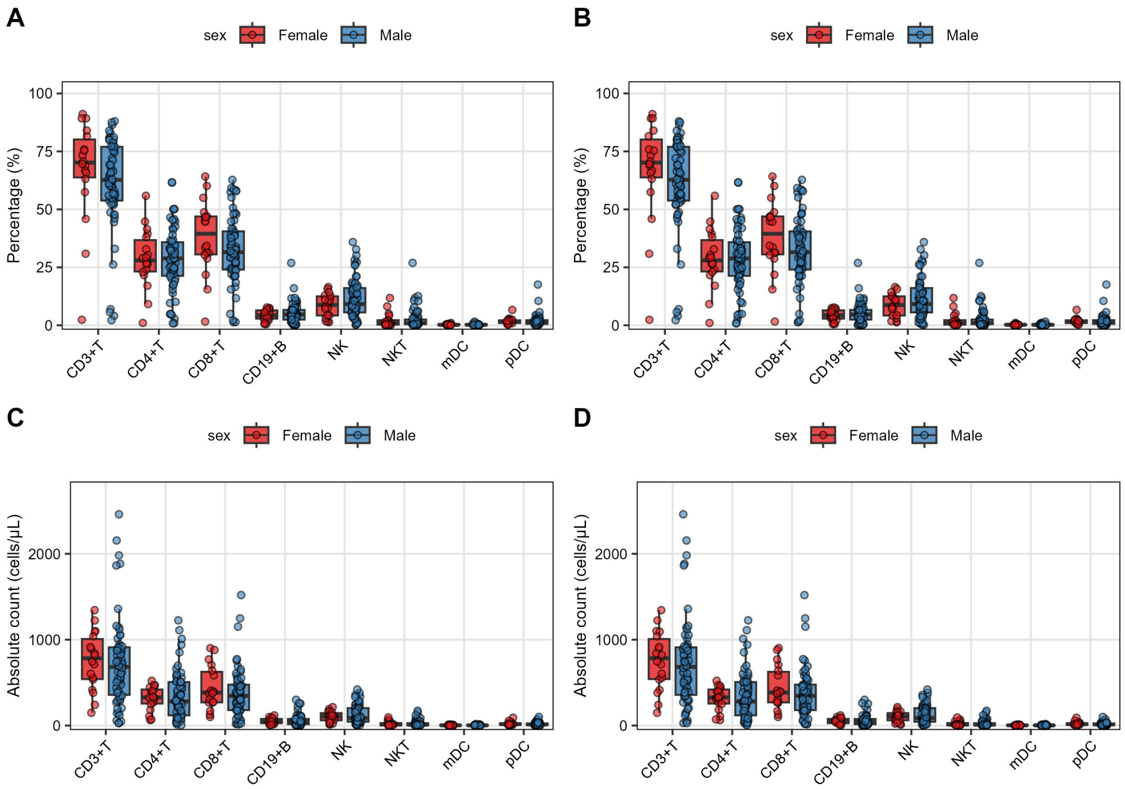

Supplementary Figure S5. UMAP visualization of post-transplant immune landscape dynamics.

Uniform Manifold Approximation and Projection (UMAP) plot showing the high-dimensional immune space of stable transplant recipients at different follow-up intervals. Points are colored by follow-up time: ≤1 month (red), 2-3 months (green), 4-6 months (blue), >6 months (purple). Early post-transplant patients (≤1 month) cluster distinctly in UMAP space (Wasserstein distance=3.21).

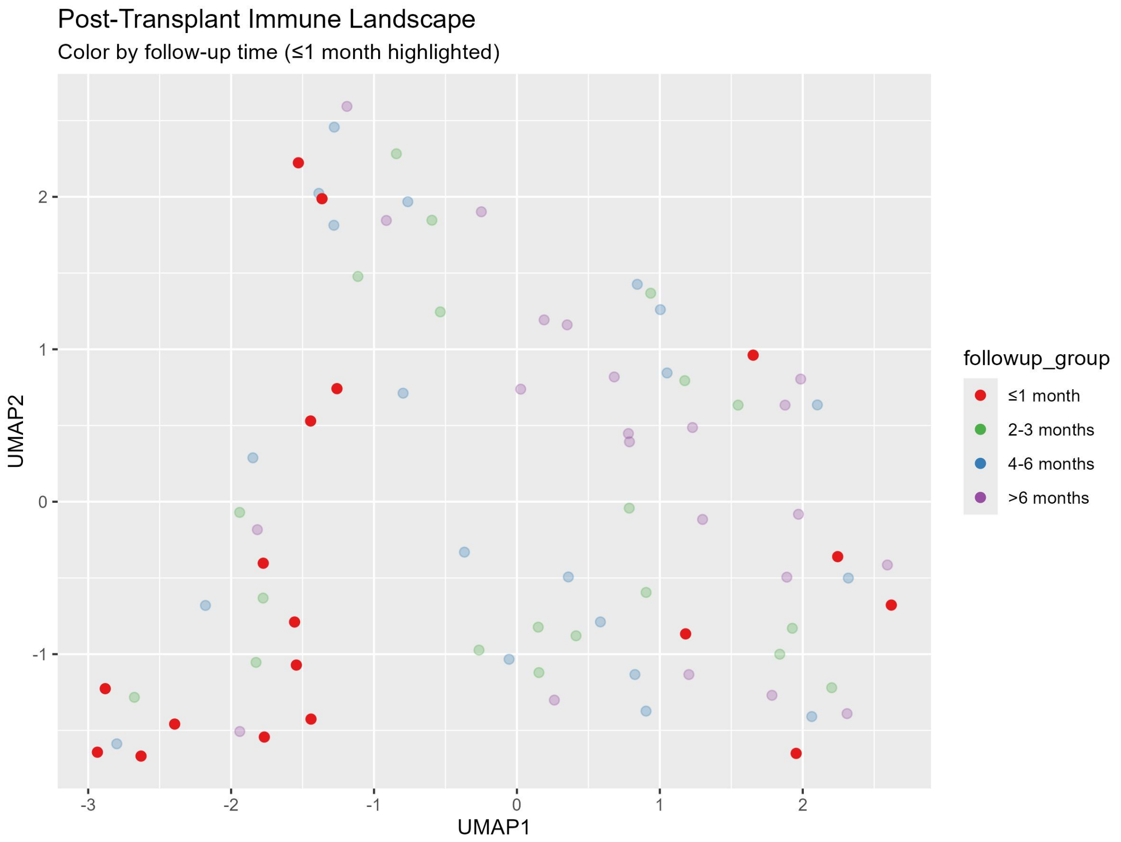

Supplementary Figure S6. Spatial immune landscape homogeneity across demographic strata.

Uniform Manifold Approximation and Projection (UMAP) visualization showing consistent immune landscapes across (A) sex, (B) age, and (C) primary liver disease subgroups in stable transplant recipients. (D) Contour density plot of ≤1 month group showing characteristic clustering pattern in UMAP space, confirming the distinctive immune state early post-transplantation.

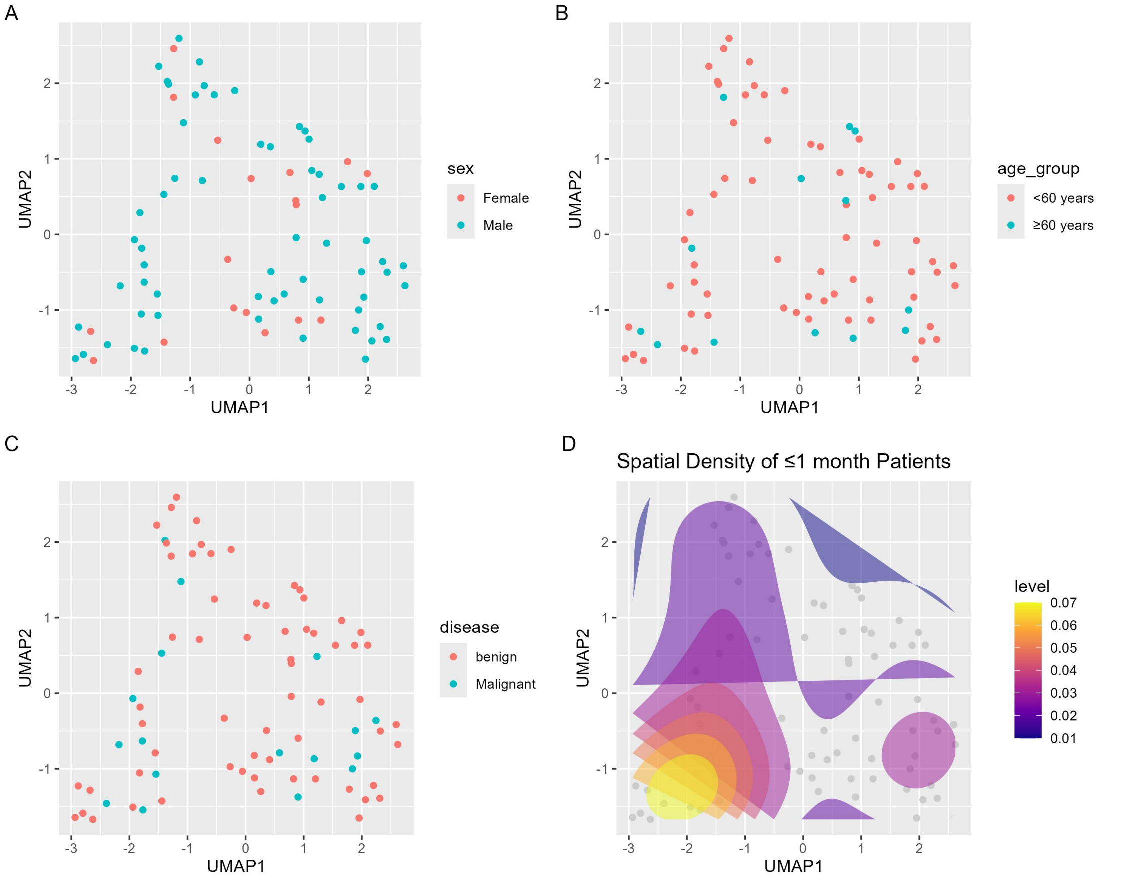

Supplementary Figure S7. Correlations among significant lymphocyte subset changes after anti-rejection therapy.

Heatmap showing Spearman correlation coefficients among Δ-changes (post-treatment minus pre-treatment) in lymphocyte subsets that showed significant alterations. Red indicates positive correlations, blue indicates negative correlations. The analysis reveals coordinated dynamics among T-cell subsets during immune recovery.

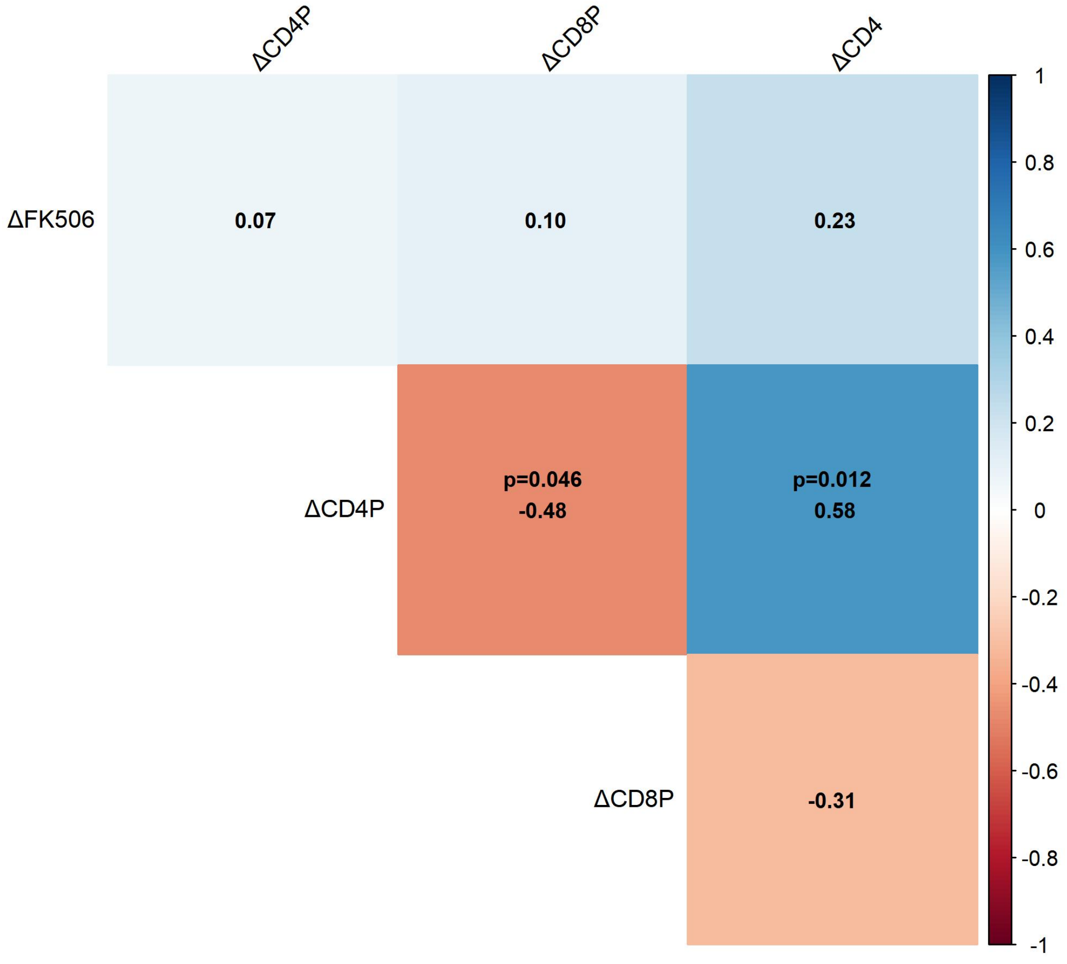

Supplementary Figure S8. Correlation plots of CD4+ and CD8+ T-cell changes after therapy.

(Left) Scatter plot showing the inverse relationship between ΔCD4+T% and ΔCD8+T% (r = -0.43, p = 0.046). (Right) Scatter plot showing the positive correlation between ΔCD4+T% and ΔCD4+T absolute count (r = 0.59, p = 0.012). These relationships indicate coupled CD4+ T-cell dynamics during anti-rejection treatment response.

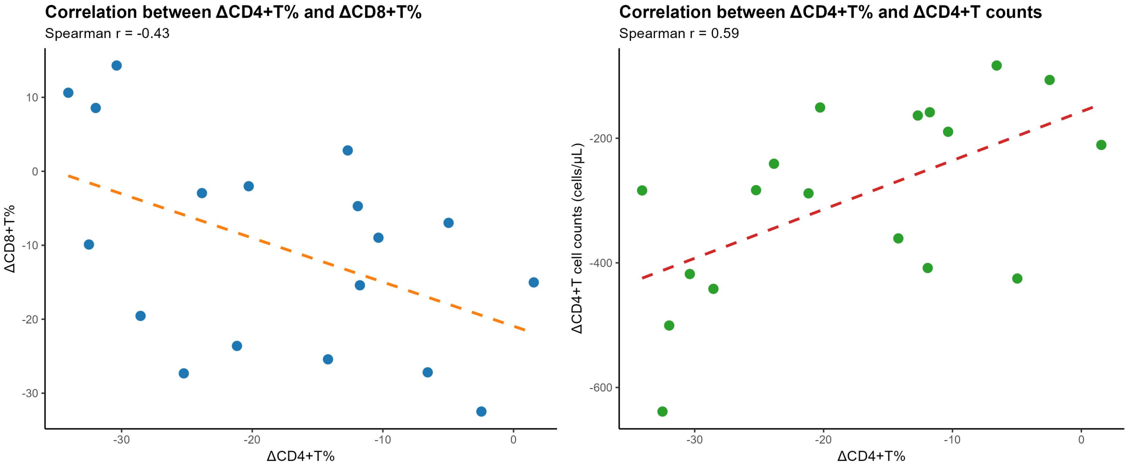

Supplementary Figure S9. Dynamic time warping (DTW) distance ranking of lymphocyte subsets.

Bar plot ranking lymphocyte subsets by their DTW distance, quantifying the temporal coherence of changes before and after anti-rejection therapy. CD3+T cell absolute count, CD4+T cell absolute count, and CD8+T cell absolute count showed the highest DTW distances (28.5, 17.9, and 10.3, respectively), indicating the most consistent reshaping following treatment.

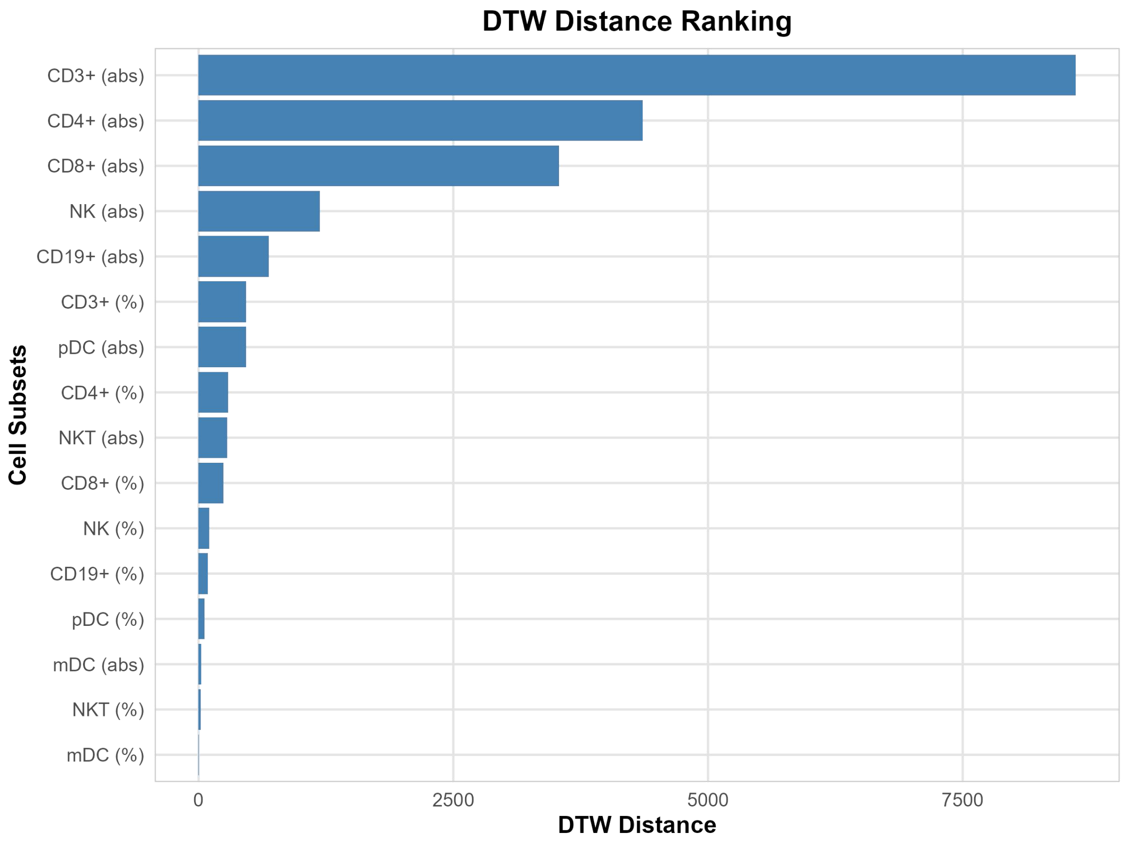

Supplementary Figure S10. Heatmap of treatment-induced changes in lymphocyte subsets.

Heatmap visualization of standardized changes (Z-scores) in all 16 lymphocyte parameters from pre- to post-treatment. Each column represents one patient (n=18), each row represents a lymphocyte parameter. Red indicates increase, blue indicates decrease. The uniform blue shift across the T-cell zone confirms synchronous suppression of T-cell subsets.

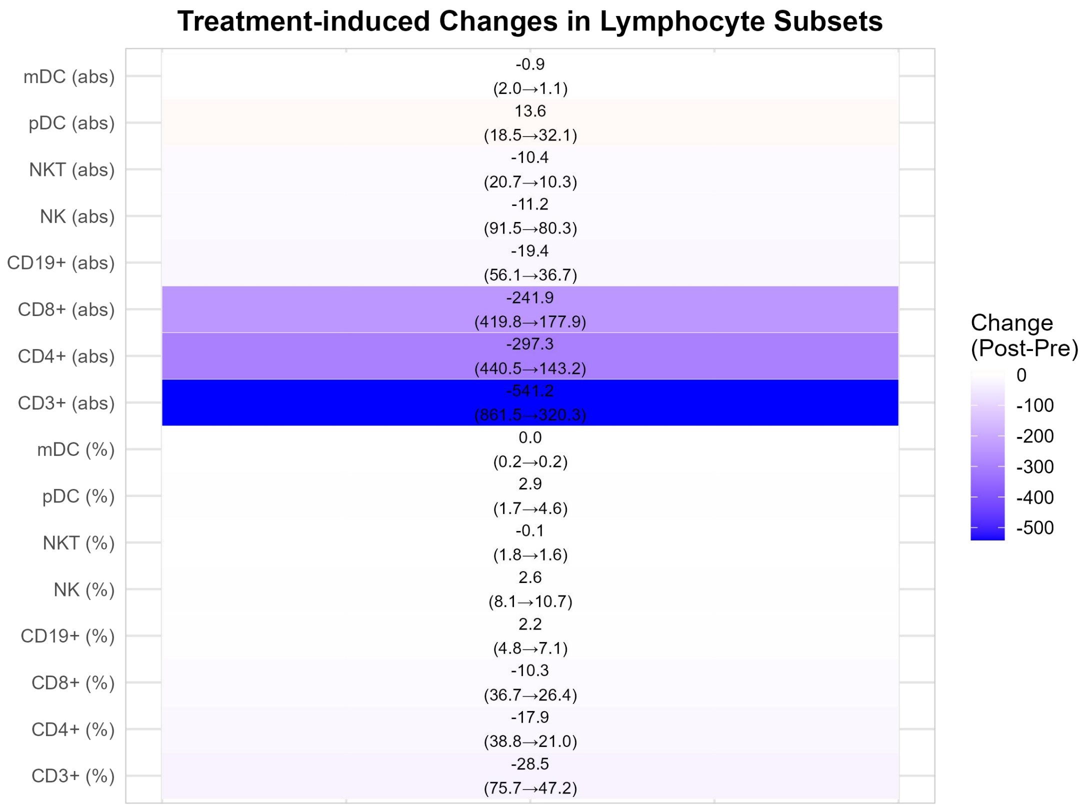

Supplementary Figure S11. Trajectory plots of the top three affected T-cell subsets.

Individual patient trajectories for (A) CD3+T cell absolute count, (B) CD4+T cell absolute count, and (C) CD8+T cell absolute count from pre- to post-treatment. Lines show tight convergence toward lower values, confirming consistent response patterns across patients.

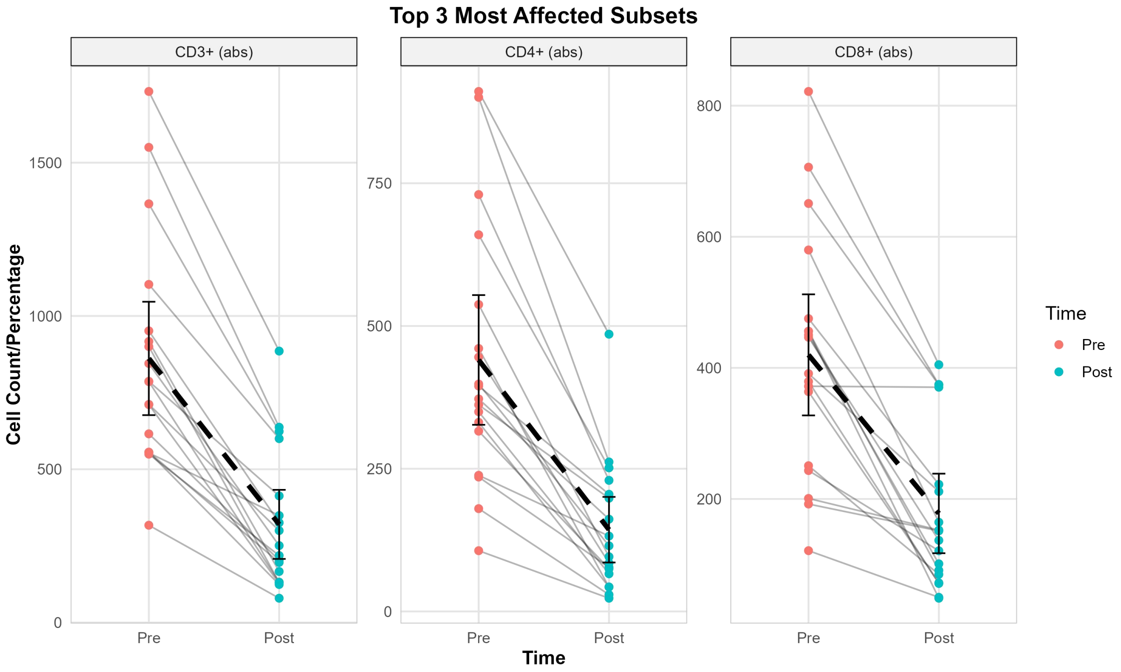

Supplementary Figure S12. Trajectories of non-significant lymphocyte subsets.

Individual patient trajectories for B cells, NK cells, and dendritic cell subsets from pre- to post-treatment. Lines show widely scattered paths with minimal consistent directionality, confirming relative preservation of these compartments following anti-rejection therapy.

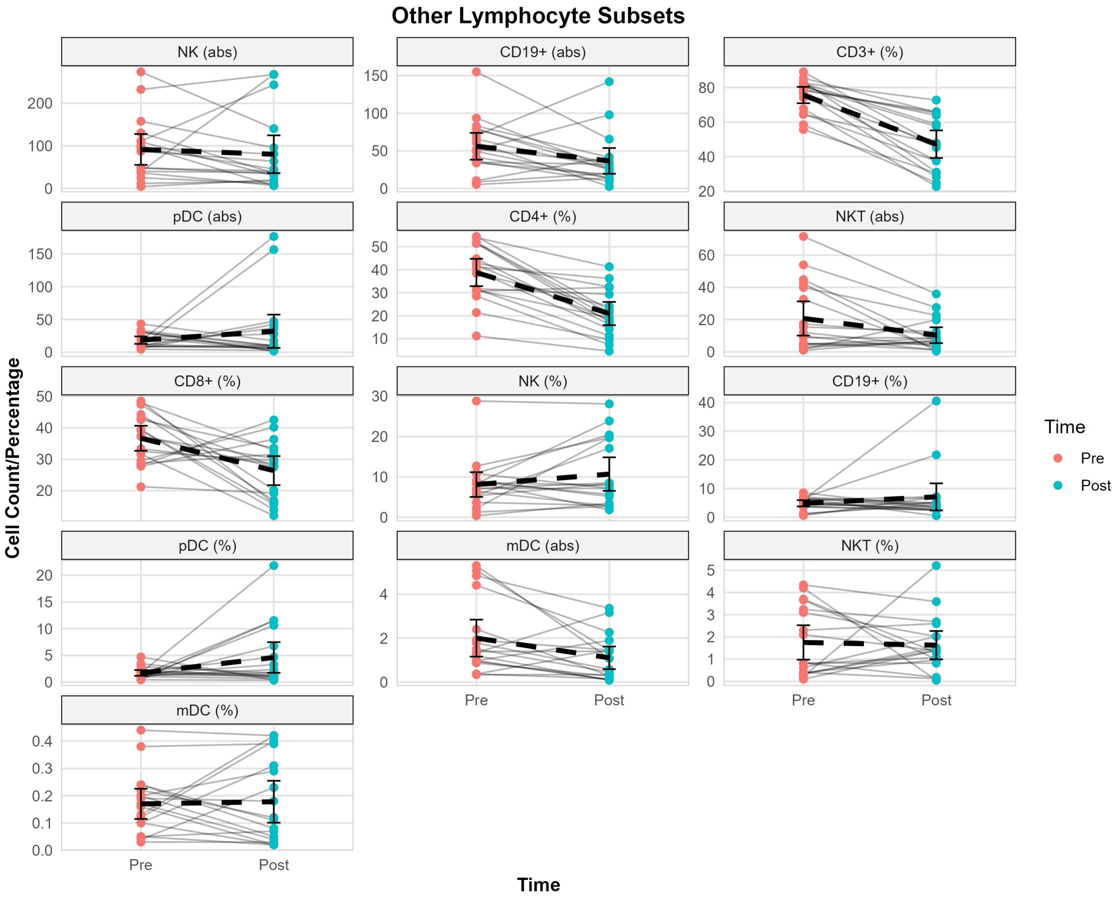

Supplementary Figure S13. Distribution differences analyzed by Wasserstein distance.

(A) Volcano plot showing effect sizes (x-axis) and -log10(FDR-adjusted q-values) (y-axis) for all lymphocyte subsets comparing non-rejection versus pre-rejection states. (B) Bar plot of top differentially abundant subsets ranked by effect size, highlighting CD3+% and CD4+% as the most differentially distributed subsets (effect sizes >1.2).

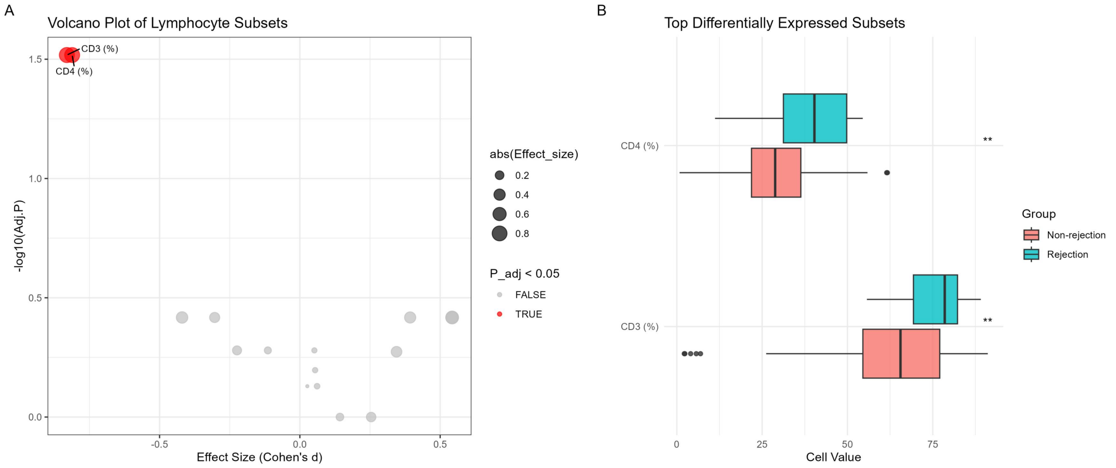

Supplementary Figure S14. Clinical characteristics comparison between groups.

Bar charts comparing sex distribution and primary liver disease (A,B) and age distribution and follow-up follow-up time (C,D) between non-rejection (n=80) and pre-rejection (n=18) groups. Statistical comparisons were performed using Fisher's exact test or Mann-Whitney U test (A,C) with Benjamini-Hochberg FDR correction (B,D). No significant differences were observed between all groups.

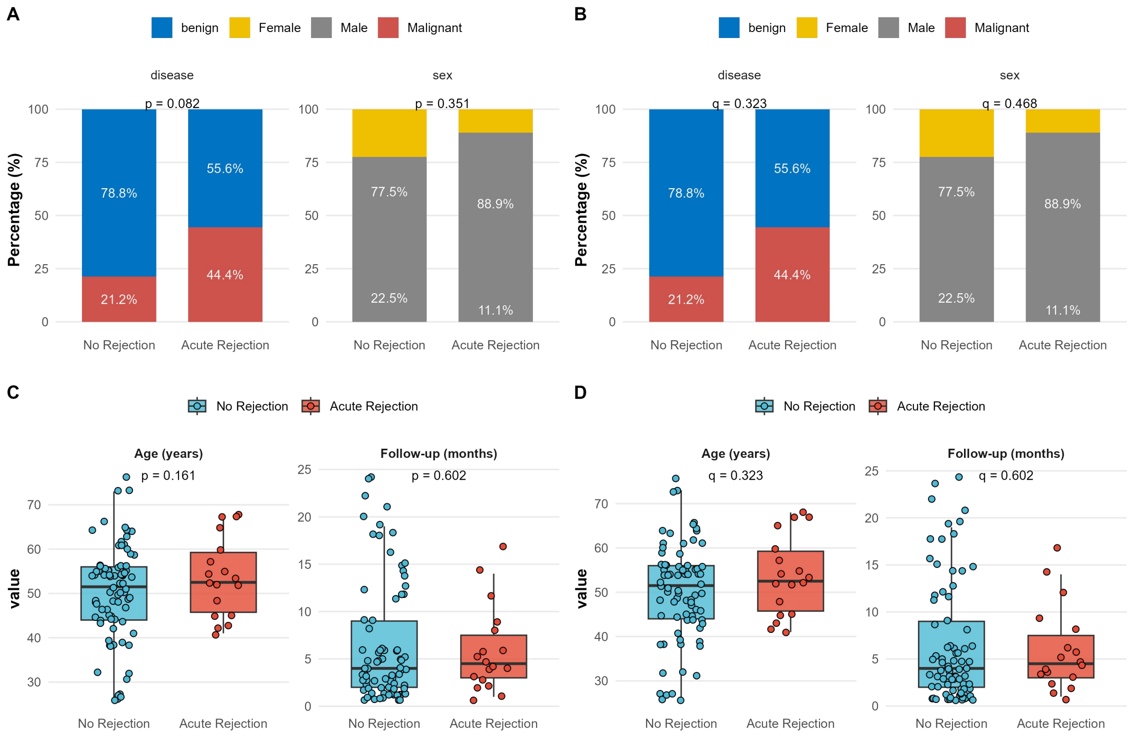

Supplementary Figure S15. Forest plot showing univariate odds ratios for all candidate predictors of acute rejection.

Odds ratios (OR) and 95% confidence intervals from Firth-penalized logistic regression analysis of individual predictors. The vertical dashed line at OR=1 indicates no association. Statistical significance was determined at p<0.05 (two-tailed). The analysis confirms FK506 level (OR=0.732, p=0.015) and CD4+ T-cell percentage (OR=1.072, p=0.005) as the most significant individual predictors, while other lymphocyte subsets showed no statistically significant association after FDR correction.

Variable	OR (95% CI)	P-value
FK506 (ng/mL)	0.73 (0.54-0.95)	0.015
CD4+ T cells (%)	1.07 (1.02-1.14)	0.005
CD3+ T cells (%)	1.04 (0.99-1.10)	0.108
CD8+ T cells (%)	1.03 (0.98-1.09)	0.175
CD19+ B cells (%)	0.98 (0.94-1.03)	0.518
NK cells (%)	0.99 (0.96-1.03)	0.798
NKT cells (%)	1.01 (0.96-1.06)	0.621
pDC (%)	1.02 (0.99-1.06)	0.227
mDC (%)	1.01 (0.98-1.05)	0.402

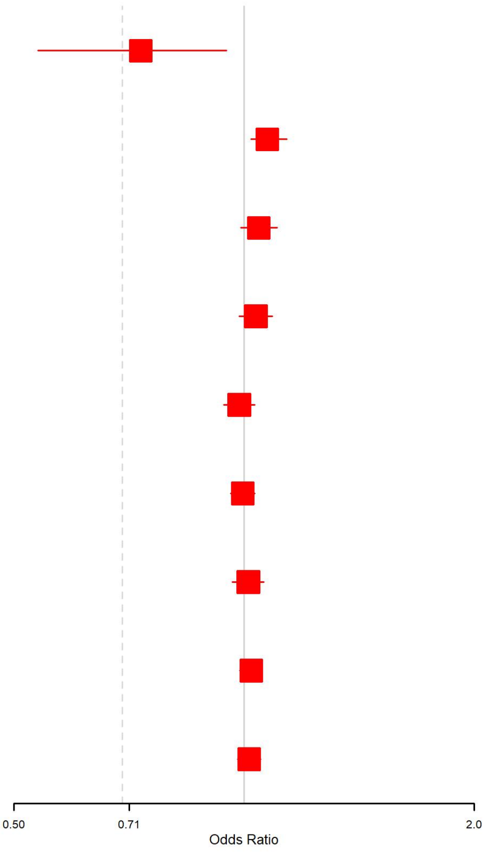

Supplementary Figure S16. Calibration plot with bootstrap correction.

Calibration curve showing the agreement between predicted probabilities and observed outcomes. The dashed diagonal line represents perfect calibration. The solid line shows the apparent calibration, while the bias-corrected line (via 500 bootstrap replicates) demonstrates good model calibration across the risk spectrum (Hosmer-Lemeshow test: χ^2=3.166, p=0.924).

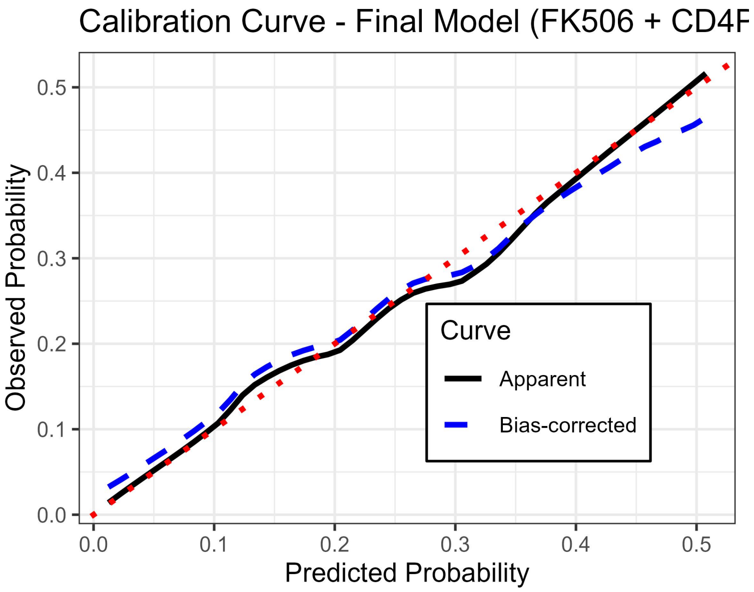

Supplementary Figure S17. Decision curve analysis demonstrating net clinical benefit.

Decision curve showing the net benefit of using the immunopharmacologic model across a range of threshold probabilities (10-50%). The model (red line) provides superior net benefit compared to treating all patients (blue line) or treating none (green line) across clinically relevant decision thresholds.

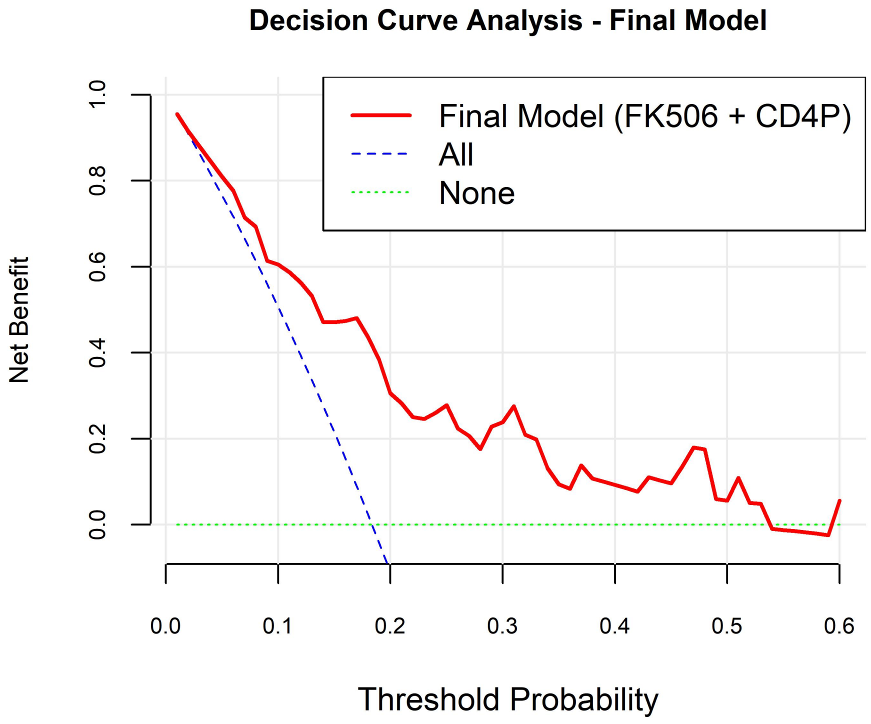

Supplementary Figure S18. Incremental value of the combined immunopharmacologic model.

Comparison of ROC curves for the combined model (FK506 + CD4P, red) versus single-parameter monitoring: tacrolimus alone (blue, AUC=0.694) and CD4+ T cells alone (green, AUC=0.733). The combined model shows statistically significant improvements over both individual components (ΔAUC=0.080, p=0.007 vs tacrolimus; ΔAUC=0.041, p=0.014 vs CD4+ T cells).

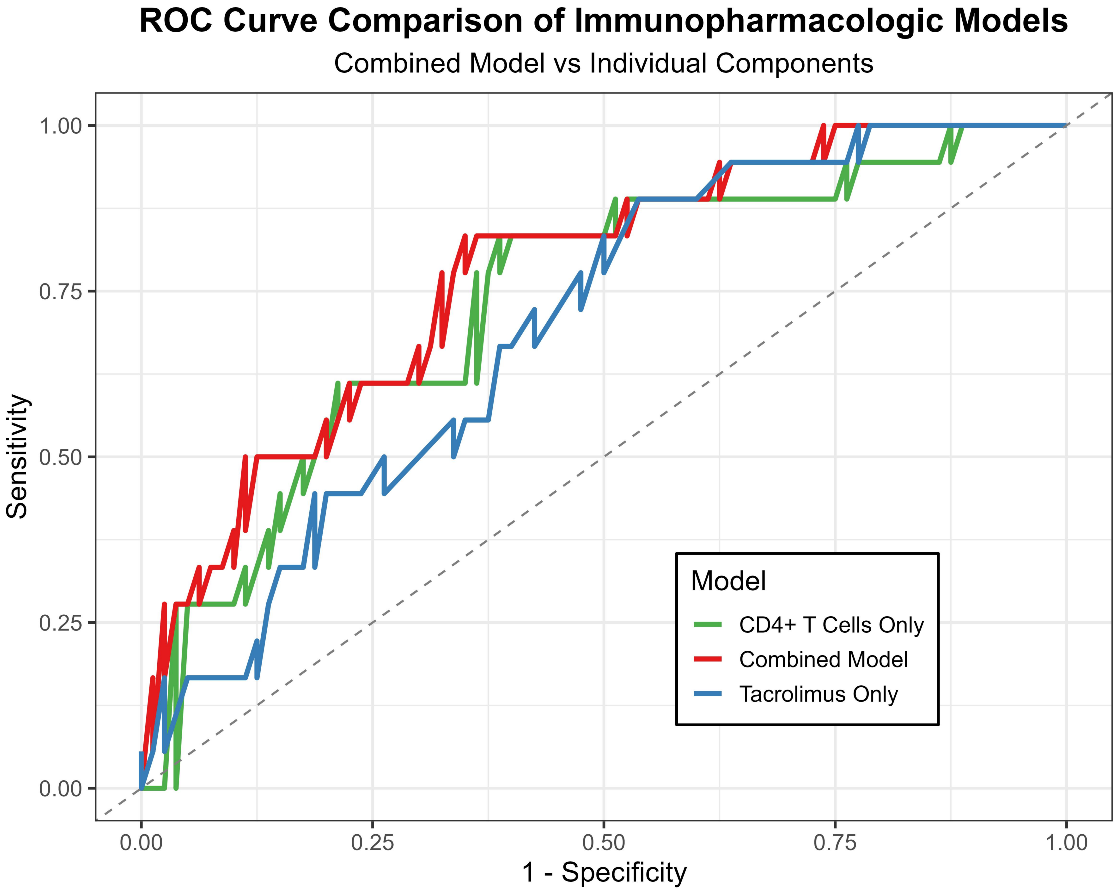

Supplementary tables

Supplementary Table S1. Comparison of Lymphocyte Subsets in Stable Liver Transplant Recipients (independent tests with FDR correction)

Supplementary Table S2. Comparison of Lymphocyte Subsets Before and After Anti-rejection Therapy (paired tests with FDR correction)

Supplementary Table S3: Summary of Δ-Changes in Lymphocyte Subsets

Supplementary Table S4: DTW Distance-Based Ranking of Lymphocyte Subsets

Supplementary Table S5: Comparison analysis of Lymphocyte Subsets between non-rejection and pre-rejection states
